# Inverse Design of Metal‐Organic Frameworks for CH_4_/N_2_ Separation Enabled by Coupled Machine Learning and Genetic Algorithms

**DOI:** 10.1002/advs.202513146

**Published:** 2025-09-19

**Authors:** Wenxuan Li, Xiaonan Zhang, Hao Guo, Lingchuan Li, Lifeng Ding, Qingyuan Yang

**Affiliations:** ^1^ State Key Laboratory of Organic‐Inorganic Composites, College of Chemical Engineering Beijing University of Chemical Technology Beijing 100029 China; ^2^ Department of Chemistry and Materials Science, Advanced Materials Research Center, School of Science Xi'an Jiaotong‐Liverpool University Suzhou Jiangsu 215123 China; ^3^ School of Chemical Engineering and Technology Xinjiang University Urumchi 830017 China; ^4^ College of Chemistry and Chemical Engineering Tarim University Alar 843300 China

**Keywords:** CH_4_/N_2_ separation, genetic algorithms, inverse design, machine learning, metal‐organic frameworks

## Abstract

Metal‐organic frameworks (MOFs) have emerged as promising candidates for gas separation, yet the vastness of their structural design space renders experimental screening prohibitively time‐ and resource‐intensive. Recent advances in machine learning (ML) technology offer powerful alternatives for accelerating MOF discovery through data‐driven prediction. In this work, a high‐accuracy ML model with a Tangent Adaptive Genetic Algorithm (TAGA) is integrated to enable inverse design of MOFs for CH_4_/N_2_ separation. The ML model, trained on structural features including topology, metal/organic secondary building units, and functional groups, is embedded within the TAGA framework to efficiently navigate the high‐dimensional chemical space. Analysis of the evolutionary trajectory reveals that MOFs featuring the **fsc** topology and ligands such as pyrene, anthracene, and naphthalene consistently exhibit superior CH_4_/N_2_ selectivity. Based on these high‐performance genotypes, a series of MOF structures are constructed, among which the top‐performing candidate achieves an IAST selectivity of 15.92 and a CH_4_ uptake of 2.47 mmol g^−1^. This study highlights a paradigm shift from trial‐and‐error screening toward goal‐directed materials design, offering a generalizable pathway for developing next‐generation separation materials.

## Graphical abstract

A machine learning‐guided genetic algorithm is employed to explore metal‐organic frameworks (MOFs) for CH_4_/N_2_ separation. By combining predictive modeling with adaptive evolution, the approach identifies high‐selectivity candidates featuring **fsc** topologies and polycyclic ligands, offering an efficient route to discovering high‐performance separation materials.

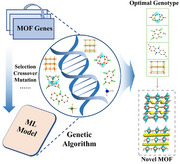

## 1 Introduction

As a new class of crystalline porous materials, metal‐organic frameworks (MOFs) are composed of metal ions or clusters that are coordinated with organic ligands in well‐defined topological architectures.^[^ ^1^ ^]^ Owing to their high surface areas, structural tunability, and chemical diversity, MOFs have emerged as promising platforms toward various application fields, including gas adsorption and separation.^[^ ^2^ ^]^ For instance, Cui et al.^[^ ^3^ ^]^ developed a copper‐based MOF with hexafluorosilicate and organic linkers designed to have a high affinity for acetylene. Cadiau et al.^[^ ^4^ ^]^ designed a fluorinated porous MOF material that selectively adsorbed propylene, with the complete exclusion of propane. Chang et al.^[^ ^5^ ^]^ synthesized SBMOF‐1, a robust calcium‐based MOF optimized for CH_4_/N_2_ separation, which uniquely accounted for the biocompatibility, low cost, low toxicity, and natural abundance of calcium. Driven by global research efforts, over 128,000 MOF structures have currently been reported and deposited in the Cambridge Structural Database (CSD),^[^ ^6^ ^]^ and this number continues to grow rapidly. While this vast structural library offers immense potential for a wide range of applications, it also renders experimental screening for application‐specific MOFs increasingly impractical.

With the advancement of artificial intelligence technology, machine learning (ML) has emerged as a transformative tool across scientific disciplines.^[^ ^7^, ^8^, ^9^ ^]^ In the field of MOFs, ML enables the use of structural and chemical descriptors to build data‐driven models that are capable of accurately predicting key properties such as gas storage capacity,^[^ ^10^, ^11^, ^12^ ^]^ separation selectivity,^[^ ^13^, ^14^, ^15^, ^16^, ^17^ ^]^ catalytic activity,^[^ ^18^ ^]^ thermal/chemical stability,^[^ ^19^ ^]^ etc. Compared to conventional experimental or simulation‐based screening approaches, ML offers a scalable and efficient alternative, facilitating rapid evaluation and optimization across large MOF datasets. Recent studies have demonstrated the growing utility of ML in accelerating MOF discovery. Lu and Li et al.^[^ ^20^ ^]^ employed ML algorithms to identify MOFs with high propane selectivity. Among the four models evaluated, the Random Forest algorithm yielded the highest predictive accuracy, ultimately leading to the identification of JNU‐90. This MOF exhibited a C_3_H_6_/C_3_H_8_ selectivity of 2.7 along with excellent recyclability. In another work, a curated set of 2,966 MOFs from the QMOF^[^ ^21^ ^]^ database was optimized using density functional theory (DFT) calculations, and subsequently used to train an equivariant neural network capable of accurately predicting energies, forces, and related properties.^[^ ^22^ ^]^ The exploration of MOF space has been significantly accelerated by the recent development of both experimental and hypothetical databases, such as CoRE‐MOF,^[^ ^23^, ^24^ ^]^ Boyd‐Woo,^[^ ^25^ ^]^ QMOF^[^ ^21^ ^]^ and ARC‐MOF,^[^ ^26^ ^]^ but current screening efforts remain confined to a narrow subspace of the vast MOF chemical design space.^[^ ^27^ ^]^ To effectively navigate the large MOF space, including hidden space that has yet to be explored, it is crucial to uncover structure‐property relationships from existing data and to implement inverse design strategies capable of generating new MOF candidates tailored to specific target properties.^[^ ^28^ ^]^ This paradigm of inverse design represents a transformative shift away from traditional trial‐and‐error methodologies, enabling rational, property‐driven discovery of next‐generation, high‐performance MOFs.

To enable inverse MOF design, genetic algorithms (GAs)^[^ ^29^ ^]^ provide an effective and versatile tool for navigating the vast MOF design space and identifying optimal candidates for specific applications.^[^ ^30^, ^31^ ^]^ Inspired by the principles of biological evolution, GAs apply iterative processes of selection, crossover, and mutation to efficiently explore vast chemical design spaces, identifying candidate materials that meet predefined performance objectives. Chung et al.^[^ ^32^ ^]^ pioneered the use of GAs to identify high‐performance MOFs for pre‐combustion CO_2_ capture from a large database.^[^ ^33^ ^]^ By screening only ∼1% of the dataset, they achieved a hundredfold increase in computational efficiency, with experimental validation confirming the predicted material performance. Collins et al.^[^ ^34^ ^]^ advanced this approach by evolving pore functional groups to enhance CO_2_ adsorption capacity. Lee et al.^[^ ^35^ ^]^ combined ML and GAs to screen trillions of hypothetical MOF structures, ultimately identifying 964 candidates with exceptional methane storage performance, 96 of which surpassed the then‐current world record. Their modular design strategy enabled the generation of novel frameworks that would be inaccessible via traditional enumeration methods. And recently, Pham and Snurr^[^ ^36^ ^]^ introduced multi‐objective optimization into the GA framework for CO_2_/N_2_ separation, yielding MOFs that outperformed existing materials across five distinct topological families and highlighting the critical role of pore size and chemical functionality in separation efficiency. A key factor in the success of GA‐based materials discovery lies in the design of the genotype, which directly influences both the predictive accuracy of the evaluation model and the breadth of chemical space explored. To date, however, no reported GA framework has employed a genotype that simultaneously encodes all three essential structural components of MOFs: topology, building blocks, and functional groups. This limitation constrains the representation of the full structural diversity of MOFs, thereby reducing the depth and diversity of the search process and hindering the identification of truly novel, high‐performance materials.

In this study, ML and GAs are integrated to accelerate the discovery of high‐performance MOFs for CH_4_/N_2_ separation, a process vital to the purification of natural gas, coalbed methane, landfill gas, and other methane‐rich sources.^[^ ^37^ ^]^ A key innovation of this work lies in the design of a comprehensive genotype that simultaneously encodes MOF topology, metal/organic secondary building units, and functional group information. These descriptors are used to construct a high‐accuracy predictive model for CH_4_/N_2_ selectivity. The model is embedded within the Tangent Adaptive Genetic Algorithm (TAGA) framework^[^ ^38^ ^]^ serving as a fitness evaluator to guide the evolutionary operations of selection, crossover, and mutation. Through iterative evolution, TAGA efficiently navigates the expansive MOF chemical space and identifies genotypes associated with superior separation performance. Subsequent statistical analysis of the evolved population reveals key structural features that contribute to enhanced CH_4_/N_2_ selectivity. Based on these representative genotypes, a series of novel MOF structures were designed, providing theoretical targets to inform experimental synthesis and validation.

## 2 Experimental Section

### 2.1 Dataset Acquisition and GCMC Simulation

The MOFs used in this work were derived from the DB0^[^ ^25^ ^]^ subset of the ARC‐MOF^[^ ^26^ ^]^ database proposed by Woo et al., comprising 203,024 structure‐validated MOFs primarily generated using the ToBasCCo^[^ ^39^ ^]^ code. The database provides high‐accuracy REPEAT^[^ ^40^ ^]^ charges for the framework atoms of each structure. To ensure the pore‐space accessibility of MOFs for gas molecules, those structures were excluded with pore limiting diameter smaller than 3.8 Å (below which CH_4_ and N_2_ molecules were unlikely to diffuse into the frameworks). Subsequently, the MOFid^[^ ^41^ ^]^ program was used to identify the SMILES^[^ ^42^ ^]^ representations of the organic building blocks of all MOFs, enabling extraction of functional group information. After removing entries with parsing or identification errors, a high‐quality dataset of 24,694 MOFs was obtained. Figure S7 (Supporting Information) presents the distribution of key geometric parameters within the final dataset, which closely mirrors the distributions observed in the original ARC‐MOF collection, thereby confirming representativeness. Each MOF structure was then encoded using six key descriptors (also called “genes”): inorganic secondary building units (SBU) that contain metal ions (Node), primary organic linkers (Linker 1), the functional groups contained in primary organic linkers (FG 1), secondary organic linkers (Linker 2), the functional groups contained in secondary organic linkers (FG 2), and topology. The full dataset encompasses 6 types of inorganic SBUs, 50 types of organic linkers, 28 types of functional groups, and 158 types of topological nets, thereby capturing an extensive and chemically diverse design space. Detailed structural information and a schematic overview of the genotype encoding strategy were provided in Figures S1–S5 (Supporting Information).

Grand Canonical Monte Carlo (GCMC)^[^ ^43^ ^]^ simulations were conducted to evaluate the competitive adsorption performance of the MOFs for equimolar CH_4_/N_2_ gas mixtures at 1.0 bar and 298 K. The TraPPE^[^ ^44^ ^]^ force field were adopted to model CH_4_ and N_2_ molecules: CH_4_ was represented as a single spherical non‐polar particle,^[^ ^45^ ^]^ while N_2_ was modeled as a three‐point molecule comprising two nitrogen atoms and a massless ghost atom positioned at the center of mass (COM).^[^ ^46^ ^]^ For MOF atoms, Lennard‐Jones (LJ) parameters were used from the DREIDING^[^ ^47^ ^]^ force field And for those elements not covered by this force field, their potential parameters were taken from the Universal Force Field (UFF).^[^ ^48^ ^]^ The accuracy of this force field combination has been validated extensively in prior studies.^[^ ^49^ ^]^ Complete parameter details were provided in Tables S2 and S3 (Supporting Information). As shown in Figures S6 (Supporting Information), the adsorption isotherms simulated for benchmark MOFs, including Cu‐BTC,^[^ ^50^, ^51^ ^]^ ZIF‐69,^[^ ^52^ ^]^ and Mg‐MOF‐74 etc.,^[^ ^53^ ^]^ exhibit close agreement with corresponding experimental data, confirming the reliability of the simulation setup. Each GCMC simulation cycle consisted of 1 × 10^6^ equilibrium cycles and 1 × 10^6^ production cycles. All simulations were carried out using this in‐house high‐throughput molecular simulation platform, HT‐CADSS.^[^ ^54^ ^]^

### 2.2 Machine Learning and Genetic Algorithm

After obtaining the genotypes of the materials and their corresponding adsorption performance, four commonly‐used ML models, were employed for data mining to uncover the structure‐performance relationships of MOFs. These models evaluated include eXtreme Gradient Boosting (XGBoost),^[^ ^55^ ^]^ Random Forest (RF),^[^ ^56^ ^]^ Support Vector Machine (SVM)^[^ ^57^ ^]^ and Linear Regression (LR),^[^ ^58^ ^]^ whose prediction performances were evaluated using three metric indicators: coefficient of determination (*R^2^*), mean absolute error (MAE) and spearman rank correlation coefficient (SRCC).^[^ ^49^, ^59^ ^]^ To interpret the contributions of individual input features, SHapley Additive exPlanations (SHAP)^[^ ^60^ ^]^ analysis was applied to the trained ML models. Based on cooperative game theory, SHAP assigns an importance value (Shapley value) to each feature by evaluating its marginal contribution to the prediction outcome across all possible feature combinations. This approach offers consistent, locally accurate, and globally interpretable insights into feature importance, enabling a detailed understanding of the structural factors influencing CH_4_/N_2_ selectivity in MOFs.

**Figure 1:**
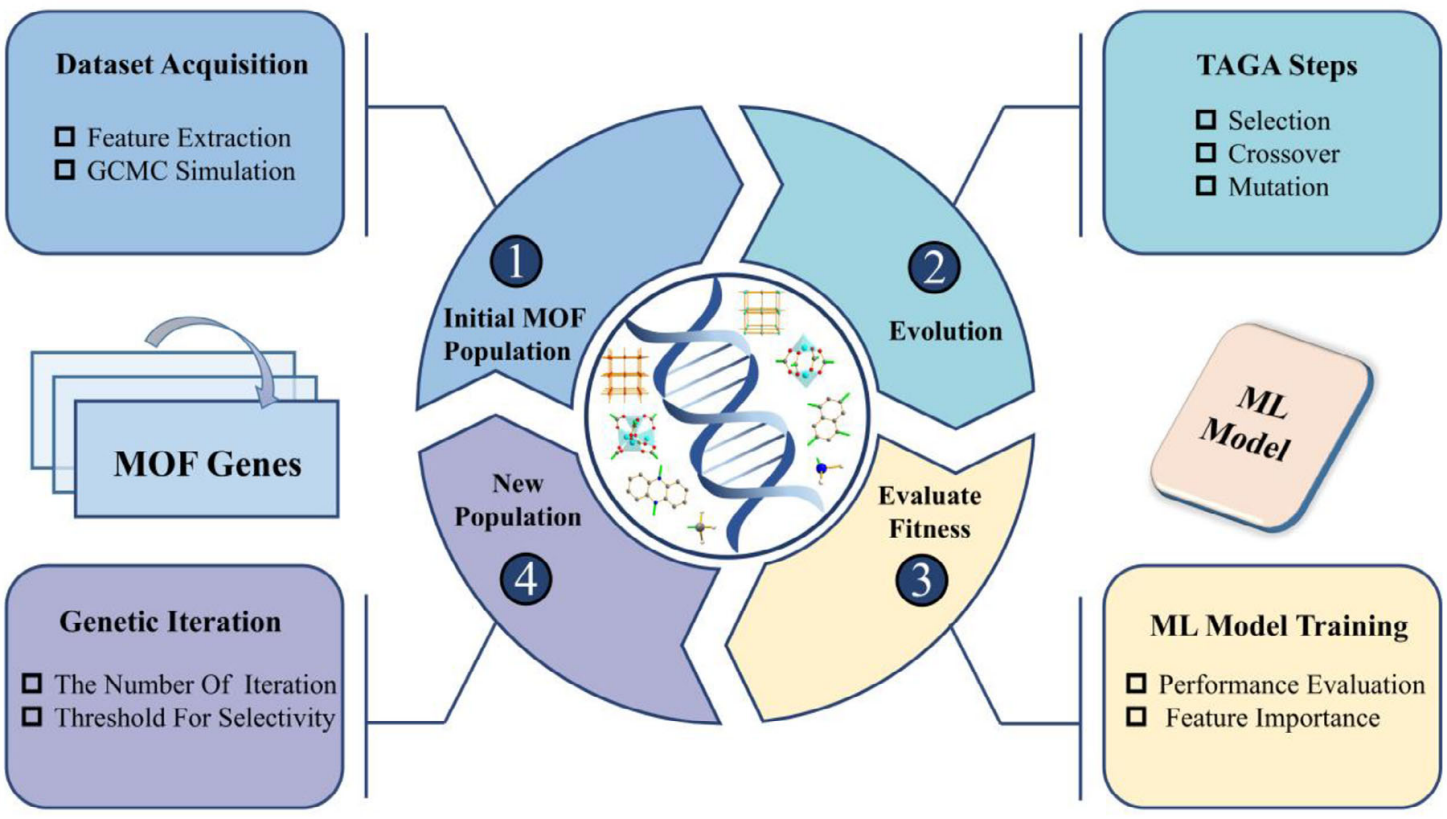
Workflow of integrating machine learning (ML) with the Tangent Adaptive Genetic Algorithm (TAGA) for the inverse design of high CH_4_/N_2_ selectivity MOF materials.

This study employed an enhanced TAGA method, which dynamically adjusts key evolutionary parameters using an exponential adaptation function. This enhancement improves search efficiency, accelerates convergence, and enhances optimization stability. Further algorithmic details were provided in Section S2 of the Supporting Information. The overall workflow was illustrated in **Figure** **1**. The process begins with feature extraction to encode each MOF structure as a genotype, followed by GCMC simulations to evaluate CH_4_/N_2_ selectivity and generate the initial dataset. The highest‐performing MOFs were selected as the initial population for genetic evolution. TAGA was then employed to iteratively evolve the genotypes through standard genetic operations: selection (retaining individuals with high fitness), crossover (recombining segments from two parent genotypes), and mutation (introducing random alterations to maintain diversity). A machine learning model served as the fitness evaluator, predicting CH_4_/N_2_ selectivity and guiding the evolutionary process by promoting genotypes with superior predicted performance. The model's predictive performance was rigorously evaluated on both training and test sets, and SHAP analysis was used to assess feature importance. Prior to genetic evolution, the total number of generations and a fitness threshold were defined; only individuals exceeding this threshold were retained for subsequent generations. In each iteration, the offspring population replaced the parent generation, and the process was repeated until convergence. Through this iterative refinement, the framework successfully identified genotypes associated with MOFs exhibiting the highest CH_4_/N_2_ selectivity.

## 3 Results and Discussion

### 3.1 Model Prediction Performance

**Figure 2:**
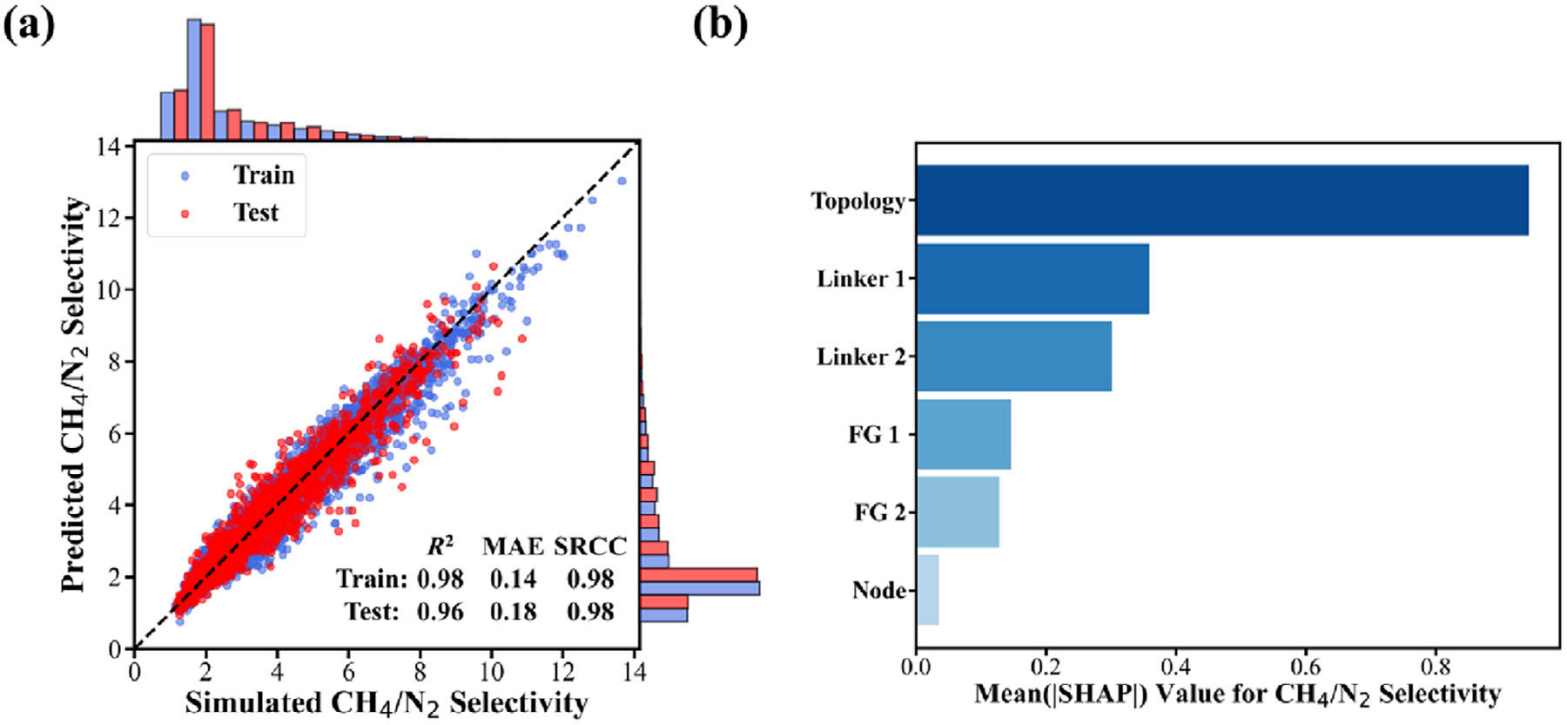
a) Prediction‐simulation scatter plots of CH_4_/N_2_ selectivity using XGBoost. The blue bars represent the data distribution of the training set, while the red bars represent the data distribution of the test set. b) Feature importance analysis of the XGBoost model for predicting CH_4_/N_2_ selectivity based on the Mean(|SHAP|) values.

Following GCMC simulations of CH_4_/N_2_ adsorption under 1.0 bar and 298 K, the CH_4_/N_2_ selectivities of the MOFs were calculated. As shown in Figure S8 (Supporting Information), all MOFs exhibit CH_4_/N_2_ selectivity greater than 1, indicating a general preference for CH_4_ adsorption. However, MOFs with high selectivity are relatively rare, with the highest selectivity observed in this simulation being 13.65. To accurately predict CH_4_/N_2_ selectivity based on structural features, four ML models were trained using the encoded genotypic data of the MOFs. The dataset was randomly split into training (80%) and test (20%) sets, with the training set used for model training and parameter optimization, and the test set for assessing predictive accuracy on unseen samples. As shown in **Figure** **2**a, the XGBoost model outperformed all others. The marginal bar plots confirm that the data distributions in the training and test sets are nearly identical, supporting the validity of the random split. The scatter plot illustrates the model's strong predictive capability on both datasets, with no evidence of overfitting. On the test set, the model achieved a *R* ^2^ of 0.96 and a SRCC of 0.98, reflecting both high accuracy and robust ranking ability. To further validate the generalization performance, 5‐fold cross‐validation was conducted on the XGBoost model. As summarized in Table S8 (Supporting Information), the results demonstrate excellent consistency across different folds. Comparative results in Tables S6 and S7 (Supporting Information) reveal that both XGBoost and RF significantly outperform SVM and LR models. Although RF and XGBoost show comparable performance on the test set, RF exhibits a clear tendency to overfit on the training data. In addition, the 5‐fold cross‐validation results for the other three models (Tables S9–S11, Supporting Information) further confirm their generalization capability and support the robustness of the XGBoost model. Considering its superior predictive accuracy, robustness, and faster inference time, XGBoost was selected as the primary machine learning model for subsequent analysis.

To assess the impact of different features on the predictive performance of the ML model, feature importance was quantified using Mean(|SHAP|) value. As shown in Figure 2b, topology emerged as the most influential feature, with a SHAP value of 0.91, substantially higher than the second‐ranked feature, Linker 1, which had a value of 0.33. This analysis reveals a clear hierarchy of feature importance: topology dominates, followed by organic linkers, functional groups, and finally the inorganic SBU. The predominance of topology reflects its central role in defining pore geometry, molecular arrangement, and confinement effects, all of which strongly influence gas adsorption and separation behavior, as demonstrated extensively in previous studies.^[^ ^49^, ^61^, ^62^ ^]^ In contrast, while organic linkers and their associated functional groups also contribute by modulating pore size and chemical environment, their influence is secondary to the overall framework topology. Once the topology is fixed, variations in the inorganic SBU have a relatively limited impact on pore morphology, which likely explains the lower importance assigned to this feature by the model.

### 3.2 Genetic Iteration Results and Optimal Structures Analysis

**Figure 3:**
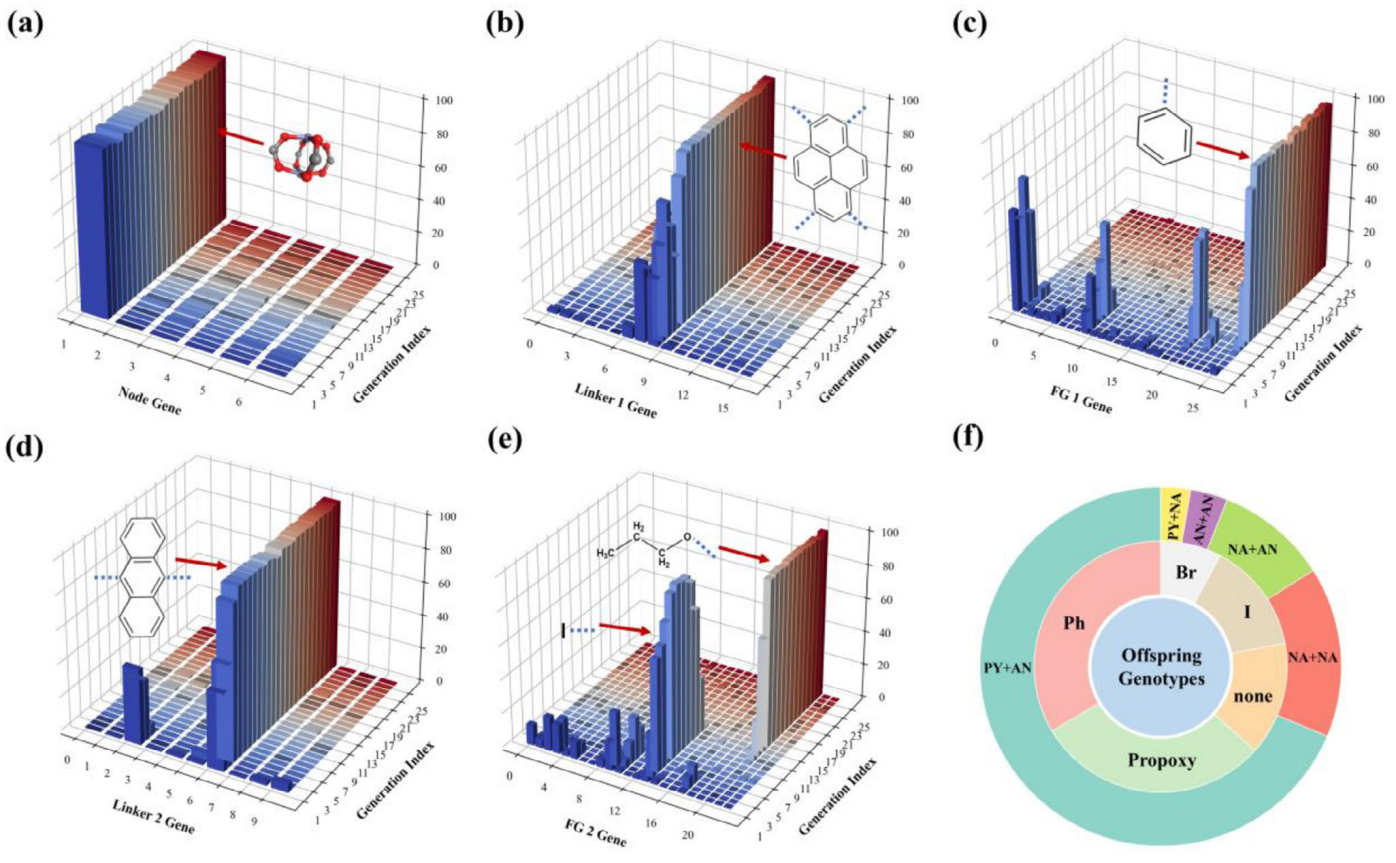
Evolution behaviors of MOF genes during the TAGA optimization process: a) Node; b) Linker 1; c) FG 1; d) Linker 2; e) FG 2. f) Dual‐ring pie chart: In offspring genotypes generated by the TAGA, the outer ring represents the combination of Linker 1 + Linker 2, and the inner ring shows the top five occurring functional group genes. (PY: pyrene, AN: anthracene, NA: naphthalene, Ph: phenyl group, I: iodo group, Br: bromo group, none: no group).

Following the completion of the ML training, XGBoost model was integrated into the TAGA framework to evaluate the fitness of each genotype based on predicted CH_4_/N_2_ selectivity. To rapidly identify high‐performing candidates, the top 100 MOFs with the highest simulated selectivities were selected as the initial parent population. A selectivity threshold of 8 was imposed to ensure that the population retained both high‐performing and structurally diverse genotypes. Figure S9 (Supporting Information) illustrates the evolution of average fitness over successive generations. The initial population exhibited an average fitness of 11, which rapidly increased to 14 within the first 10 generations. In subsequent generations, the average fitness plateaued with minor fluctuations due to crossover and mutation events. To thoroughly explore the fitness landscape, the TAGA process was extended to 200 generations. **Figure** **3**a–e track the evolution of individual genotype components over the first 25 generations. Notably, all offspring during this period retained the same **fsc** topology; thus, no variation was observed, and its trend is omitted from the figures. Given that previous SHAP analysis identified topology as the most influential factor in CH_4_/N_2_ selectivity, the persistence of the **fsc** net highlights its critical role in identifying high‐performance MOFs. Similarly, the inorganic SBUs (Node) exhibited minimal variation, with zinc paddlewheel clusters dominating throughout the evolutionary process (Figure 3a). However, given that the model identified Node as a relatively unimportant feature, this lack of diversity had little impact on the final performance. Figure 3b–e reveal that the linker and functional group genes initially displayed a variety of favorable variants, which progressively converged toward a dominant configuration: Linker 1 stabilized as pyrene, FG 1 as phenyl, Linker 2 as anthracene, and FG 2 evolved from iodine in early generations to propoxy in later stages.

To further assess the diversity of genotypes generated by the TAGA framework, duplicate entries were removed from the full set of offspring, resulting in a total of 219 unique genotypes. Analysis of ligand combinations within these structures, as shown in Figure 3f, reveals a strong preference for pyrene as the four‐connected linker and anthracene as the two‐connected linker. Across the dataset, pyrene, anthracene, and naphthalene emerge as the most frequently used organic linkers in high‐performing MOFs. Notably, in the genotype encoding scheme, ligands with differing numbers of connection sites are treated as distinct genotypes, even if their chemical identity is otherwise identical. For example, anthracene with two versus four connection points is assigned to separate genotype classes. Interestingly, optimal individuals generated by the genetic algorithm often incorporate both forms, indicating that TAGA can recognize and exploit underlying chemical similarities between genotypically distinct but chemically related ligands. This suggests that the evolutionary process is not merely confined to explicit structural rules, but is capable of capturing latent chemical relationships. Within the **fsc** topology framework, MOFs incorporating pyrene, anthracene, and naphthalene as organic linkers demonstrate higher potential for CH_4_/N_2_ selectivity. As for functional groups, phenyl, propoxy, and iodine appear most frequently among high‐selectivity genotypes. However, given the relatively low feature importance of functional groups in the predictive model, these observations should be interpreted as suggestive rather than definitive.

The TAGA workflow developed in this study yielded 27 novel genotypes with predicted selectivities surpassing those of the best‐performing MOFs in the original dataset. Based on these genotypes, corresponding MOF structures were manually constructed and subsequently subjected to DFT geometry optimization using the CASTEP^[^ ^63^ ^]^ module in Materials Studio. Atomic charges were calculated using the PACMAN^[^ ^64^ ^]^ machine learning method, which has been widely adopted for charge assignment in MOF structures.^[^ ^24^ ^]^ **Table** **1** presents the top ten newly generated MOFs ranked by their CH_4_/N_2_ selectivities obtained from GCMC simulations. The highest CH_4_/N_2_ selectivity reached 15.74, consistent with the top predicted selectivity from the XGBoost model, thereby validating the model's capability in identifying high‐performance MOFs. The MOFs adsorption potential (△*Q*) and ideal adsorbed solution theory (IAST) selectivities were further calculated. The detailed procedures for geometry optimization and separation performance calculations are provided in Section S4 of the Supporting Information. The top‐performing MOFs were also evaluated for synthetic accessibility and stability. All ligands used in their construction have synthetic accessibility (SA)^[^ ^65^ ^]^ and synthetic complexity (SC)^[^ ^66^ ^]^ scores below commonly accepted synthetic feasibility thresholds,^[^ ^67^ ^]^ and the predicted thermal^[^ ^68^ ^]^ and water stabilities^[^ ^69^ ^]^ of the MOFs are within acceptable ranges (Tables S13 and S14, Supporting Information), supporting the experimental realizability of these structures. Notably, among the two MOFs with simulated CH_4_/N_2_ selectivities exceeding 15, the second‐ranked MOF (genotype 1‐32‐18‐18‐11‐2) exhibited slightly lower selectivity than the top‐ranked material in GCMC simulations. However, its adsorption potential (2.85 mol L^−1^) was significantly higher. It is further noted that the two high‐selectivity MOFs differ only in FG2, being an iodine (I) group in one case and a methyl (CH_3_) group in the other. As shown in Table S15 (Supporting Information), the iodine‐substituted MOF exhibits a slightly higher isosteric heat of adsorption at infinite dilution ($Q_{\mathrm{st}}^{0}$) for CH_4_, which indicates its stronger CH_4_–framework interactions, but its larger steric size reduces the pore volume from 0.21 cm^3^ g^−1^ (methyl) to 0.12 cm^3^ g^−1^ (iodine). Consequently, CH_4_ uptake decreases from 2.14 to 0.97 mmol g^−1^. Further analysis showed that the second‐ and fourth‐ranked MOFs differ only in Linker 1: naphthalene in the former and pyrene in the latter. Despite similar pore volumes and porosities, the naphthalene‐based MOF exhibits slightly higher CH_4_ uptake, with a $\Delta Q_{\mathrm{st}}^{0}$ of 6.77 kJ mol^−1^ compared to 6.33 kJ mol^−1^ for the pyrene‐based MOF (Table S15, Supporting Information). These results indicate that, under this genotype configuration, naphthalene as Linker 1 provides stronger gas discrimination, leading to better CH_4_/N_2_ separation. Based on these findings, the second‐ranked MOF (genotype 1‐32‐18‐18‐11‐2) was selected for subsequent in‐depth analysis.

**Table 1:** CH_4_/N_2_ separation performance of top 10 generated MOFs.

Rank	Genotype of MOFs	△*Q* [mol L^−1^]	S_IAST_	S_XGBoost_	S_GCMC_
1	1‐32‐18‐18‐17‐2	1.74	17.47	13.78	15.74
2	1‐32‐18‐18‐11‐2	2.85	15.92	13.60	15.21
3	1‐34‐9‐18‐17‐2	2.33	6.93	13.45	14.78
4	1‐34‐18‐18‐11‐2	2.42	14.51	13.52	14.36
5	1‐34‐4‐18‐17‐2	2.63	19.87	13.15	13.88
6	1‐34‐18‐18‐17‐2	2.20	13.64	13.59	13.82
7	1‐34‐18‐18‐10‐2	2.35	13.96	13.52	13.64
8	1‐34‐3‐18‐17‐2	2.51	13.46	13.07	13.29
9	1‐32‐10‐18‐17‐2	2.44	12.50	13.22	12.66
10	1‐34‐18‐18‐24‐2	1.58	12.19	13.68	12.41

**Figure 4:**
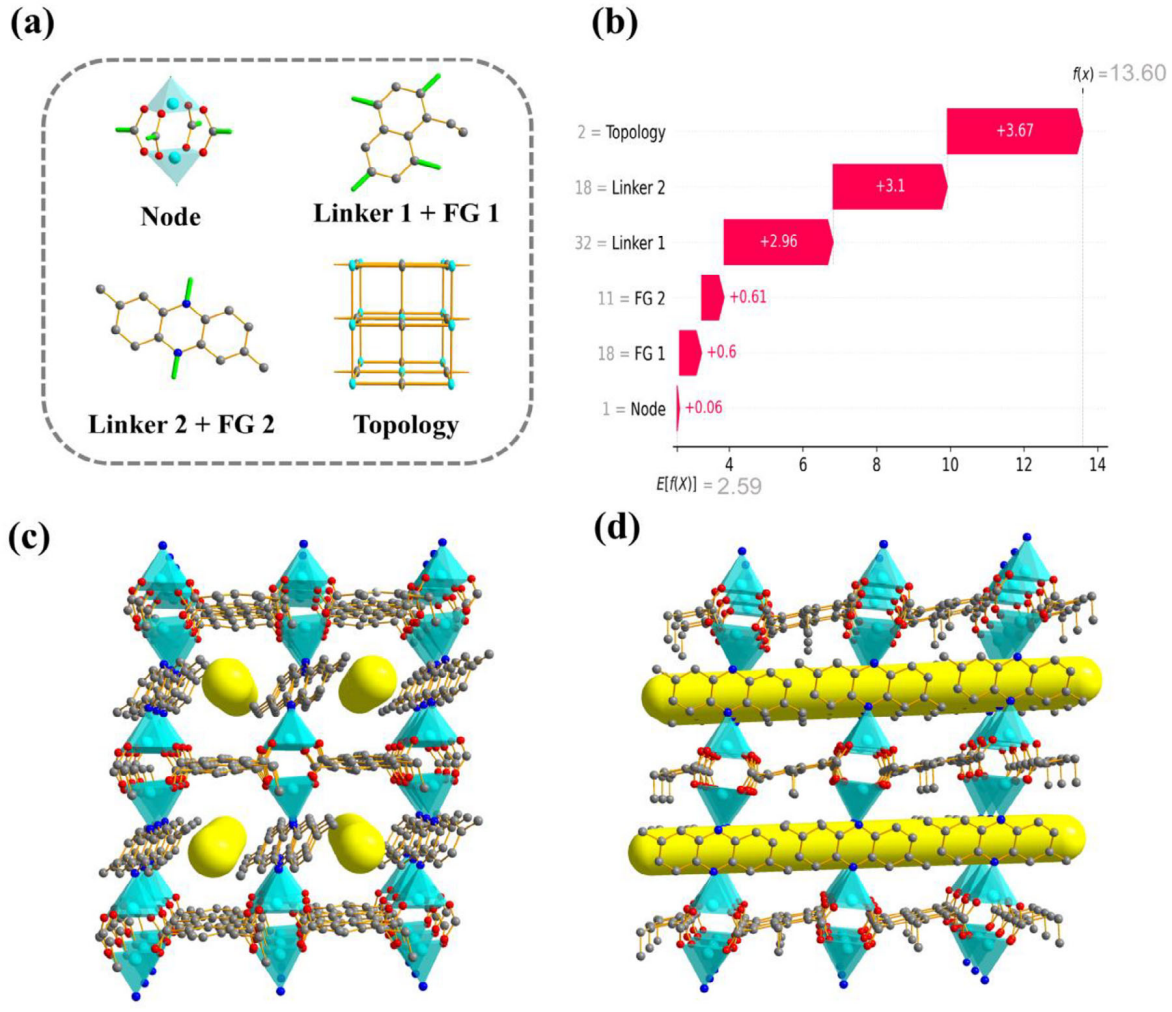
a) Schematic illustration of the topology and building blocks of the optimal MOF. b) SHAP waterfall plot of the optimal MOF's CH_4_/N_2_ selectivity prediction using the XGBoost model. Crystal structures of the optimal MOF: c) front view, d) side view.

**Figure** **4**a illustrates the structural building units of the optimal MOF identified in this study. The framework adopts an **fsc** topology, with the inorganic node comprising a zinc paddlewheel secondary building unit. The primary organic linker is a functionalized naphthalene derivative, specifically, 1‐ethylnaphthalene, which serves as the four‐connected node within the framework. The secondary organic linker is derived from anthracene, whose coordination sites are directly bonded to metal centers and thus substituted with nitrogen atoms to form the final linker: 2,7‐dimethylphenazine. Figure 4b presents a SHAP analysis quantifying the contribution of each gene in the optimal genotype to the model's predicted CH_4_/N_2_ selectivity. The baseline prediction of the XGBoost model, representing the average selectivity across the entire dataset, is 2.59. All genotype components in the optimal MOF contribute positively to the final prediction. Notably, topology has the largest individual impact (+3.67), followed by Linker 2 (+3.10) and Linker 1 (+2.96). Although the contributions of the functional groups and the inorganic SBU are relatively modest, they still enhance the overall prediction. Collectively, these features yield a predicted selectivity of 13.60, which is in close agreement with the GCMC simulated value of 15.21, validating the accuracy of the ML‐guided design approach. As shown in Figure 4c,d, the final structure features zinc paddlewheel clusters linked via two 1‐ethylnaphthalene and two 2,7‐dimethylphenazine ligands, coordinated through carboxylate groups and nitrogen atoms, respectively. This assembly gives rise to a three‐dimensional **fsc** topology network with a 6+4+2 connectivity scheme, forming well‐defined, rectangular one‐dimensional channels that facilitate selective gas transport and adsorption.

**Figure 5:**
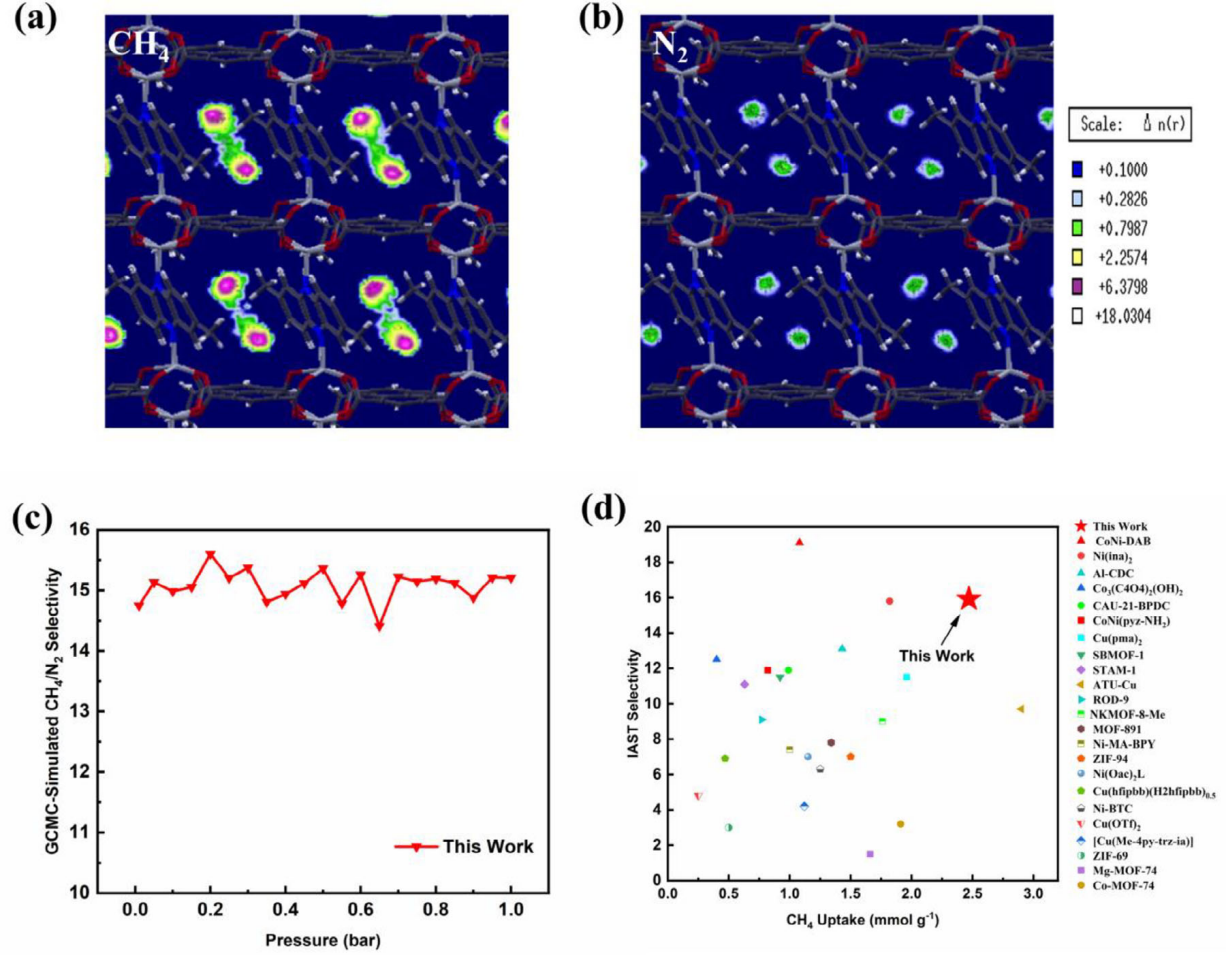
2D color‐contour plots of the center of mass (COM) probability densities of adsorbed a) CH_4_ and b) N_2_ in the optimal MOF. c) GCMC‐Simulated CH_4_/N_2_ selectivity of the optimal MOF under 0–1 bar, 298K, CH_4_/N_2_ (5:5, v/v). d) Comparison of IAST selectivity of CH_4_/N_2_ vs. amount of CH_4_ uptake for previously reported MOFs with this work.

To investigate the preferred adsorption sites in the optimal MOF, 2D contour plots of the number density for the center of mass (COM) probability distributions of adsorbed CH_4_ and N_2_ were calculated based on GCMC simulation results. As shown in **Figure** **5**a,b, the highest adsorption density for both CH_4_ and N_2_ is concentrated at the center of the one‐dimensional channels. However, the CH_4_ density is markedly higher than that of N_2_, suggesting that the framework geometry facilitates favorable interactions between CH_4_ molecules and the functional groups decorating the inner channel surfaces. This cooperative effect significantly enhances CH_4_ adsorption capacity. Notably, previous experimental studies have reported similar high CH_4_/N_2_ selectivities in MOFs featuring narrow one‐dimensional microchannels.^[^ ^70^, ^71^ ^]^ Figure 5c shows the simulated CH_4_/N_2_ selectivity of the optimal MOF as a function of pressure in the range of 0–1 bar. The MOF maintains consistently high selectivity across the entire pressure range, with values ranging from 14.41 to 15.6, demonstrating its robustness under varying conditions. As illustrated in Figure 5d, the material also achieves an excellent balance between CH_4_ uptake and IAST selectivity. Specifically, it exhibits a CH_4_ uptake of 2.47 mmol g^−1^, second only to ATU‐Cu (2.90 mmol g^−1^),^[^ ^72^ ^]^ while achieving an IAST selectivity of 15.92, second only to CoNi‐DAB,^[^ ^73^ ^]^ and among the highest reported for experimentally studied materials to date.

## 4 Conclusion

This study presents an integrated ML and GAs workflow for the inverse design of MOFs tailored for CH_4_/N_2_ separation. By incorporating key structural features, including topology, inorganic nodes, organic linkers, and functional groups, a high‐accuracy machine learning model was developed to predict CH_4_/N_2_ selectivity, achieving an *R* ^2^ of 0.96 on the test set and demonstrating excellent generalization performance. Feature importance analysis highlighted topology as the dominant factor governing selectivity, followed by linker identity and functionality. Building upon this foundation, the trained model was embedded within the TAGA as a fitness evaluator, enabling the generation of novel high‐performance genotypes through iterative evolutionary processes. Analysis of the evolved genotypes revealed that MOFs featuring **fsc** topology and linkers based on pyrene, anthracene, and naphthalene consistently exhibited superior selectivity. Structural realization of these genotypes led to the identification of an optimal MOF comprising an **fsc** net, zinc paddlewheel inorganic units, and 1‐ethylnaphthalene and 2,7‐dimethylphenazine as organic linkers. This MOF exhibits excellent separation performance, with an IAST selectivity of 15.92 and a CH_4_ uptake of 2.47 mmol g^−1^. Further analysis revealed that the 1D channel architecture of the optimal MOF promotes CH_4_ adsorption through cooperative interactions while maintaining stable separation performance across a wide pressure range. Overall, this work not only identifies promising new candidates for CH_4_/N_2_ separation but also demonstrates the power of inverse design in uncovering structure‐property relationships, offering valuable guidance for both theoretical exploration and experimental realization of next‐generation gas separation materials.

## Conflict of Interest

The authors declare no conflict of interest.

## Supporting information



Supporting Information

## Data Availability

The Python code as well as data used in this work can be found on GitHub: https://github.com/lwx199906/MOF‐TAGA.
