# Supplementary material for: Inverse Design of Metal‐Organic Frameworks for CH4/N2 Separation Enabled by Coupled Machine Learning and Genetic Algorithms
Source: Adv Sci (Weinh). 2025 Sep 19;12(46):e13146. doi: 10.1002/advs.202513146 (PMC12697905; doi:10.1002/advs.202513146)
Supplement: Supplementary file 1 — Supporting Information [file ADVS-12-e13146-s001.docx]

Supporting Information

Inverse Design of Metal-Organic Frameworks for CH_4_/N_2_ Separation Enabled by Coupled Machine Learning and Genetic Algorithms

Wenxuan Li, Xiaonan Zhang, Hao Guo, Lingchuan Li, Lifeng Ding,* and Qingyuan Yang*

**Table of Contents**

Section S1. Genetic information of the database……………………………………………S2

Section S2. Principle of the TAGA algorithm………………………………………………S5

Section S3. Details of model training …………………………………………………………S7

S3.1. Machine learning algorithms……………………………………………………S7

S3.2. Model performance metrics……………………………………………………S7

S3.3. Shapley additive explanation……………………………………………………S8

Section S4. Computational methods for MOFs……………………………………………S10

S4.1. LJ parameters…………………………………………………………………S10

S4.2. Geometric optimization methods………………………………………………S11

S4.3. Calculations of IAST selectivity………………………………………………S11

S4.4. Calculations of separation potential……………………………………………S11

S4.5. Calculations of adsorption heat………………………………………………..S12

S4.6. Calculations of pore volume…………………………………………………S12

Section S5. GCMC simulation results………………………………………………………S13

Section S6. Evaluation of machine learning and TAGA……………………………………S15

Section S7. Details of the Top-performance MOFs…………………………………………S18

References…………………………………………………………………………………S23

**Section S1. Genetic information of the database**


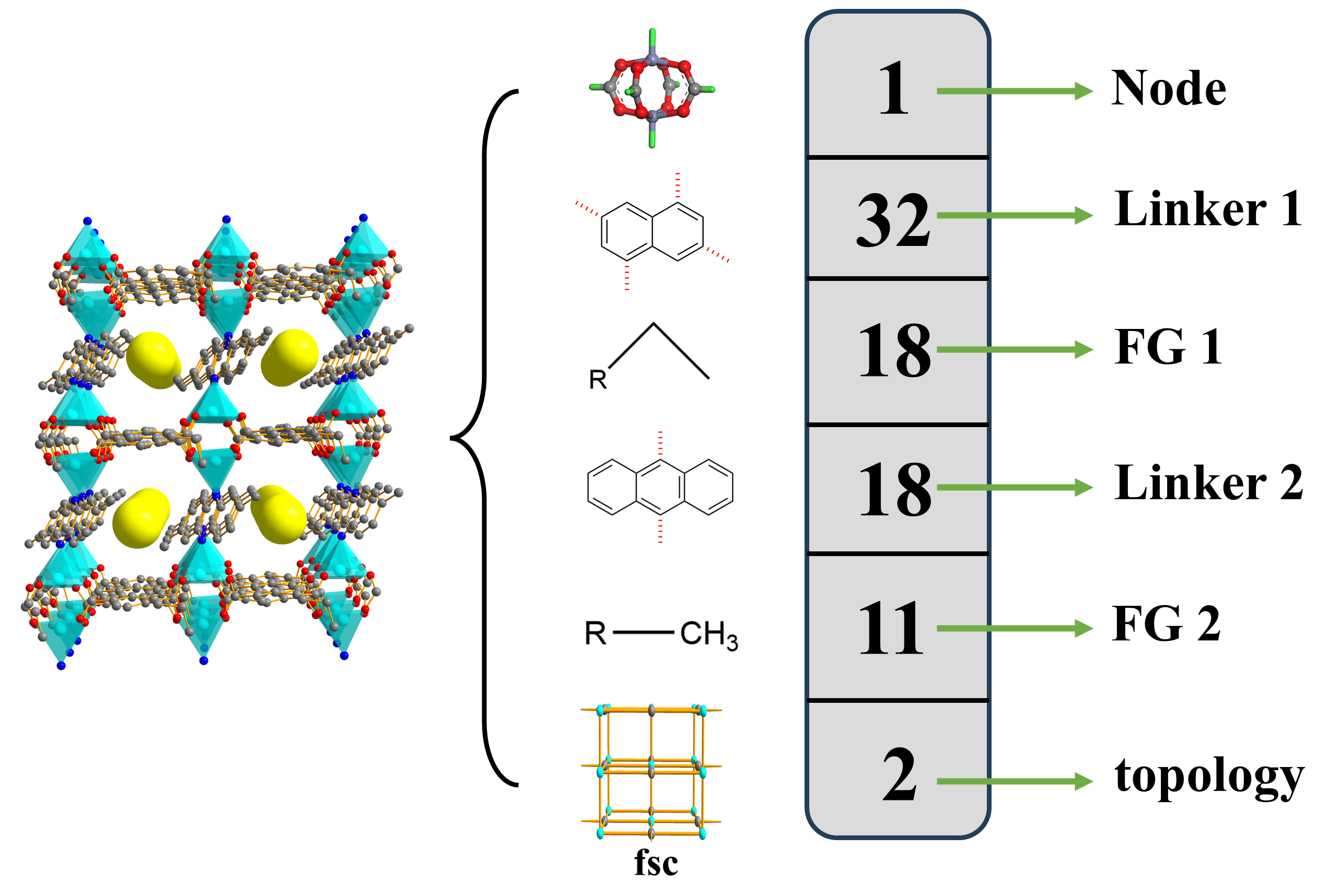


**Figure S1.** An example scheme of the gene encoding for each MOF in the database.


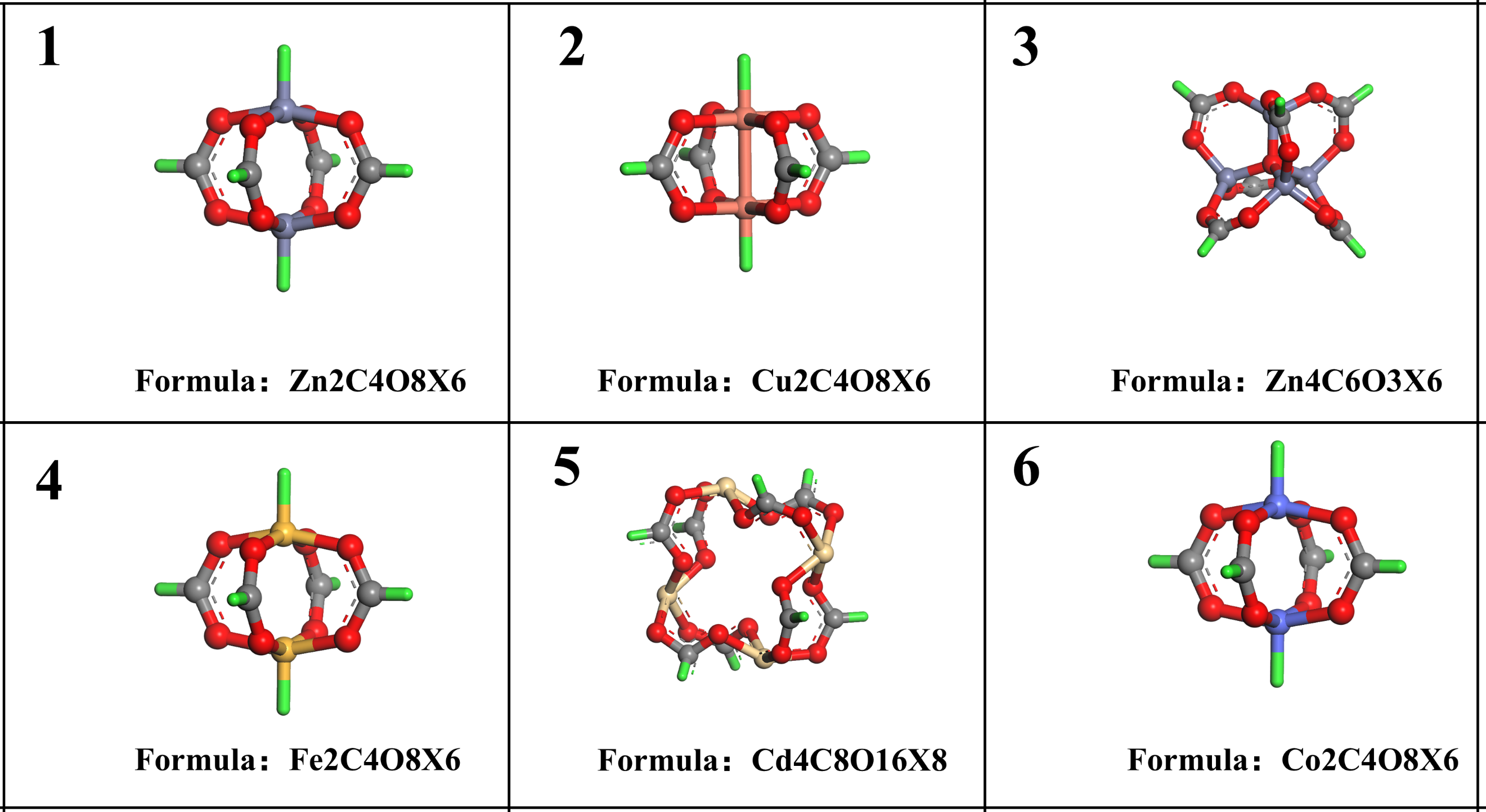


**Figure S2.** Schematic diagram of the inorganic secondary building unit used in this work. The green sticks represent connection sites.


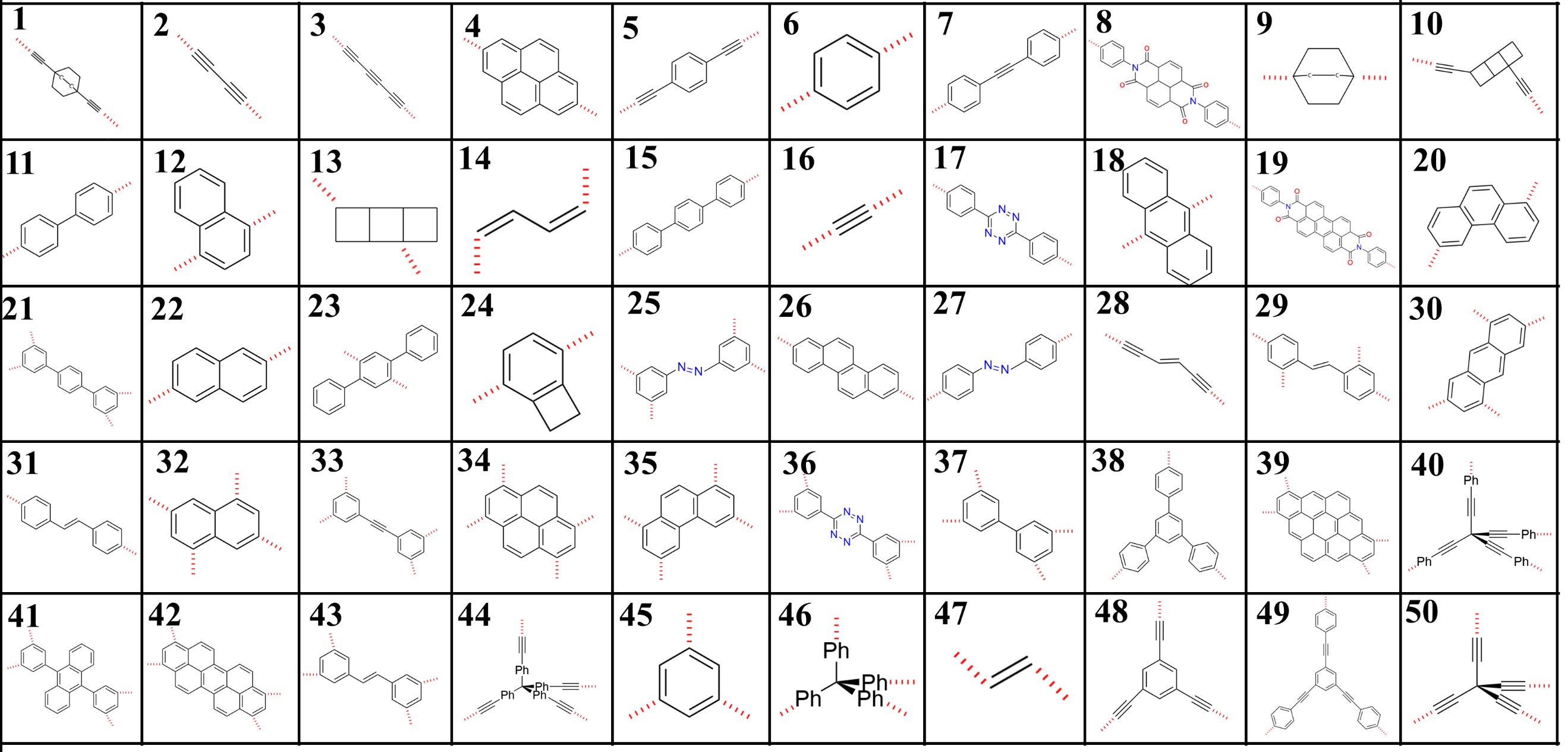


**Figure S3.** Schematic diagram of the organic ligands used in this work.


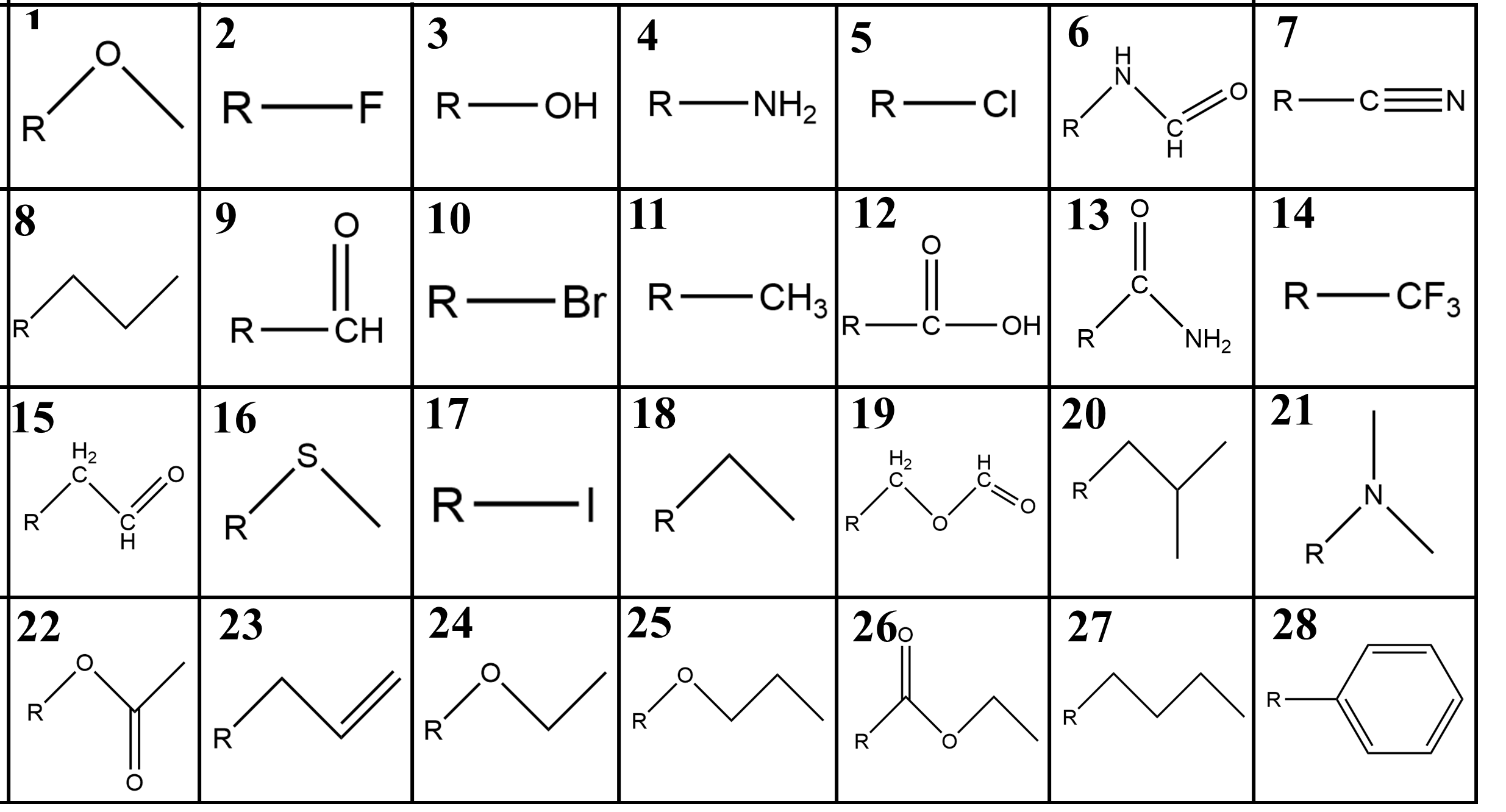


**Figure S4.** Schematic diagram of the functional groups used in this work.


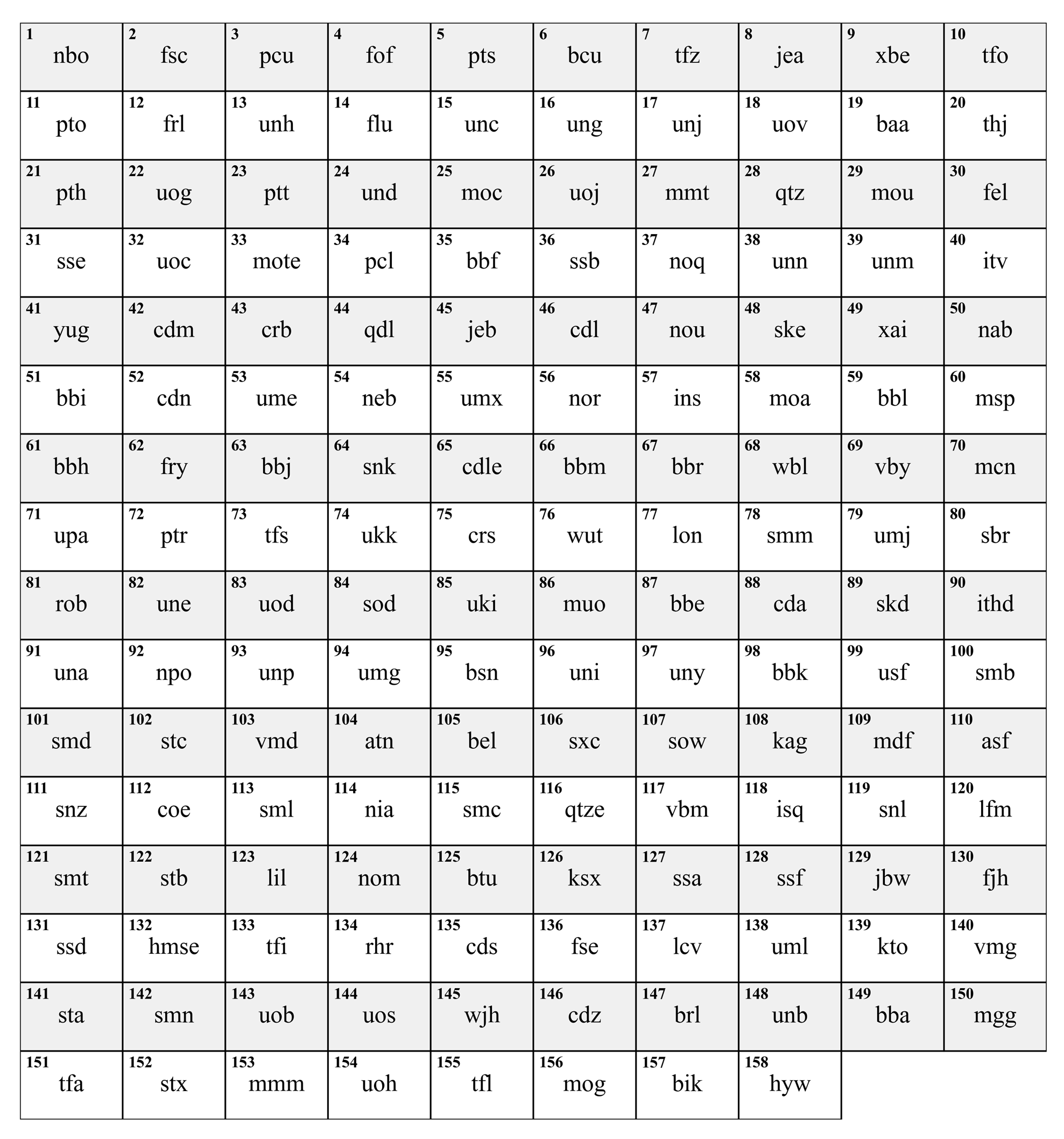


**Figure S5.** All topologies used in this work.

**Section S2. Principle of the TAGA algorithm**

In the Tangent Adaptive Genetic Algorithm (TAGA)^[1]^, each generation’s evolution process primarily includes three core operations: selection, crossover, and mutation. First, after generating the initial offspring, four candidate individuals are randomly selected from the population. Based on the specified selection rate (*P*_s_), the fittest individual is typically retained; if this condition isn’t met, the second-best individual is chosen to ensure the preservation of either the best or second-best genes. Next, the crossover rate (*P*_c_) determines whether a crossover will occur: if it does, a random crossover point is generated, with the first segment of genes taken from parent 1 and the second segment from parent 2, thereby creating a new offspring genotype. If no crossover occurs, the fitter parent is directly chosen as the child. During mutation, with a probability equal to the mutation rate (*P*_m_), a random gene position is selected, and a new value is randomly chosen from the permissible range of that gene excluding its current value to ensure a genuine mutation.

Studies have shown that the *P*_c_ and *P*_m_ have the most significant influence on the performance of genetic algorithms. Large *P*_c_ and *P*_m_ values tend to disrupt the fittest chromosomes in the later stages of evolution, causing the algorithm to behave more like a purely random search. Conversely, small *P*_c_ and *P*_m_ values can avoid this disruption but often lead to premature convergence and local optima. To address these challenges, TAGA incorporates an adaptive adjustment strategy that dynamically updates *P*_c_ and *P*_m_ based on an exponential function, expressed as follows:

$P_{\text{c}}=\left\{\begin{matrix} p_{\text{c,min}}+(p_{\text{c,max}}-p_{\text{c,min}})\frac{4}{\exp[A\times(f_{avg}-f')]+\exp[-A\times(f_{avg}-f')]+2} & f'\geq f_{avg} \\ P_{c,\max} & f'<f_{avg} \end{matrix}\right.$ (1)

$P_{\text{m}}=\left\{\begin{matrix} p_{\text{m},\min}+(p_{\text{m},\max}-p_{\text{m},\min})\frac{4}{\exp[A\times(f_{avg}-f')]+\exp[-A\times(f_{avg}-f')]+2} & f'\geq f_{avg} \\ P_{\text{m},\max} & f'<f_{avg} \end{matrix}\right.$ (2)

here, *P*_c,max_ and *P*_c,min_ represent the maximum and minimum crossover rates, while *P*_m,max_ and *P*_m,min_ denote the maximum and minimum mutation rates. *A* represent the adaptive coefficient. The variable *f* ′ represents the larger fitness value of the two parents selected for crossover, *f* is the fitness value of the individual to be mutated, and *f*_avg_ is the average fitness of the population in the current generation. According to this adaptive strategy, when *f* ′ and *f* are close to *f*_avg_, *P*_c_ and *P*_m_ take on larger values, which increases the likelihood of evolving individuals with medium fitness and thereby enhances population diversity in the early evolution stage. Conversely, when *f* ′ and *f* differ greatly from *f*_avg_, *P*_c_ and *P*_m_ become smaller, favoring the preservation of the fittest individuals. This self-adaptive and smoothly tangent-shaped adjustment of *P*_c_ and *P*_m_ helps improve both the convergence speed and precision of the genetic algorithm. All hyperparameters used for TAGA in this study are listed in Table S1.

**Table S1.** Parameters of TAGA.

| Parameters | Values |
| --- | --- |
| *A* | 0.002 |
| *P*_s_ | 0.95 |
| *P*_c,max_ | 0.65 |
| *P*_c,min_ | 0.4 |
| *P_m_*_,max_ | 0.075 |
| *P_m_*_,min_ | 0.05 |

**Section S3. Details of model training**

**S3.1.** **Machine learning algorithms**

To comprehensively evaluate the predictive performance of different machine learning models in this study, four representative models were trained on the same dataset, and the best-performing one was selected. The models include Linear Regression (LR)^[2]^, Support Vector Machine (SVM)^[3]^, Extreme Gradient Boosting (XGBoost)^[4]^, and Random Forest (RF)^[5]^.

Linear regression is a simple yet efficient model that builds the relationship between independent and dependent variables based on a linear combination of features. Its advantages include ease of interpretation, fast training speed, and suitability for large-scale datasets.
SVM is a powerful supervised learning algorithm widely used for both classification and regression tasks. It works by finding an optimal hyperplane in the feature space that maximizes the margin between different classes. SVM is particularly effective for handling high-dimensional, small-sample, and structurally complex datasets. XGBoost is an ensemble learning algorithm based on gradient boosting, which constructs multiple decision trees and combines them to form a strong predictive model. It excels at capturing nonlinear relationships in structured data and has demonstrated outstanding performance in numerous data science competitions. Random Forest is another ensemble approach that consists of multiple decision trees. During training, it randomly samples both features and data instances to build each tree, and aggregates their predictions through averaging or voting. This method offers strong robustness, especially for high-dimensional data, complex feature interactions, and datasets with missing values.

All machine learning models used in this study were trained and evaluated using their default hyperparameter settings. The training and evaluation processes were conducted on the same dataset to ensure the comparability and fairness of the results. The models were implemented using the Python libraries Scikit-learn and XGBoost.

**S3.2. Model performance algorithms**

The performance evaluation metrics used in this study are *R*^2^, MAE, and SRCC.Their calculation expressions are given as follows:

$R^{2}=1-\frac{\frac{1}{M}\sum_{m=1}^{M} (\overline{y}-\widehat{y})^{2}}{\frac{1}{M}\sum_{m=1}^{M} (y-\widehat{y})^{2}}$ (3)

$\text{MAE}=\frac{\sum_{m=1}^{M} \left|y-\widehat{y}\right|}{M}$ (4)

$\text{SRCC}=1-\frac{6\sum D^{2}}{\text{n}(n^{2}-1)}$ (5)

where them, *M* represents the sample size, *y* and $\hat{y}$ represent the actual and predicted values, and $\bar{y}$ represents the average of the actual values. SRCC calculates the rank correlation between actual and predicted values, where D represents the difference in ranks and n represents the number of observations. *R*^2^ measures the model’s global explanatory power, MAE provides an intuitively interpretable average error metric, and SRCC focuses on the consistency of prediction rankings, making it particularly suitable for material screening and prioritization.^[6, 7]^

k-fold cross-validation^[8]^ is a method used to evaluate model performance. The dataset is evenly divided into *k* subsets; each time, one subset is used as the test set and the remaining *k−1* subsets are used for training. This process is repeated *k* times, and the average result is taken to assess the model’s stability and generalization ability. In this work, *k =* 5 is used.

**S3.3.** **Shapley additive explanation**

This study employs the SHAP (Shapley Additive Explanations)^[9]^ method to interpret the importance and contribution of different predictors in the model analysis. SHAP is a game theory-based explanatory approach that represents the model’s prediction as the sum of the contribution values of all input features. When approximating the original model *f* for a specific input *x*, the attribution values *φ*_i_ for each feature *i* are required to sum up to the model output *f*(*x*), as shown in Equation (6):

(6)

The sum of the attribution values *φ*_i_(*f*,*x*) for all features equals the output of the original model *f*(*x*), where *n* denotes the total number of input features. *φ*_0_ represents the expected value of the model output when all inputs are missing, while *φ*_i_ quantifies the specific contribution of feature *i* to the prediction. According to game theory, the Shapley value is the only attribution method that satisfies the properties of local accuracy, missingness, and consistency. SHAP values are also highly interpretable, as they share the same units as the model output (TSP in this study). Essentially, a SHAP value is the Shapley value of the conditional expectation function *f*(*x*), which can be derived from Equation (7):

(7)

where *R* denotes the set of all possible feature orderings, and $P_{i}^{R}$ represents the set of features that precede feature *i* in a given ordering *R*. *M* is the total number of input features in the model. For tree-based models, this study employs the Tree Explainer algorithm proposed by Lundberg et al.^[10]^ to efficiently compute SHAP values. The algorithm calculates SHAP values individually for each sample in the dataset and then aggregates and visualizes the results to provide a global interpretation of the model.

**Section S4. Computational methods for MOFs**

**S4.1. LJ parameters**

**Table S2.** LJ parameters used to describe CH_4_ and N_2_.

| Absorbates | Atoms | ε/K | σ/Å |
| --- | --- | --- | --- |
| CH_4_ | single sphere | 148 | 3.73 |
| N_2_ | N_N2 | 36 | 3.31 |
|  | COM_N2 | 0 | 0 |

**Table S3.** LJ parameters for framework atoms.

| Atom | ε/K | σ/Å | Atom | ε/K | σ/Å | Atom | ε/K | σ/Å |
| --- | --- | --- | --- | --- | --- | --- | --- | --- |
| Ac | 16.61 | 3.10 | Ge | 201.30 | 3.80 | Pr | 5.03 | 3.21 |
| Ag | 18.12 | 2.81 | H | 7.65 | 2.85 | Pt | 40.26 | 2.45 |
| Al | 156.01 | 3.91 | He | 28.18 | 2.10 | Pu | 8.05 | 3.05 |
| Am | 7.05 | 3.01 | Hf | 36.23 | 2.80 | Ra | 203.31 | 3.28 |
| Ar | 93.10 | 3.45 | Hg | 193.75 | 2.41 | Rb | 20.13 | 3.67 |
| As | 206.33 | 3.70 | Ho | 3.52 | 3.04 | Re | 33.21 | 2.63 |
| At | 142.92 | 4.23 | I | 256.66 | 3.70 | Rh | 26.67 | 2.61 |
| Au | 19.63 | 2.93 | In | 276.79 | 4.09 | Rn | 124.81 | 4.25 |
| B | 47.81 | 3.58 | Ir | 36.74 | 2.53 | Ru | 28.18 | 2.64 |
| Ba | 183.18 | 3.30 | K | 17.61 | 3.40 | S | 173.12 | 3.59 |
| Be | 42.78 | 2.45 | Kr | 110.71 | 3.69 | Sb | 276.79 | 3.88 |
| Bi | 260.68 | 3.89 | La | 8.56 | 3.14 | Sc | 9.56 | 2.94 |
| Bk | 6.54 | 2.98 | Li | 12.58 | 2.18 | Se | 216.40 | 3.59 |
| Br | 186.20 | 3.52 | Lr | 5.54 | 2.88 | Si | 156.01 | 3.80 |
| C | 47.86 | 3.47 | Lu | 20.63 | 3.24 | Sm | 4.03 | 3.14 |
| Ca | 25.16 | 3.09 | Md | 5.54 | 2.92 | Sn | 276.79 | 3.98 |
| Cd | 114.74 | 2.54 | Mg | 55.86 | 2.69 | Sr | 118.26 | 3.24 |
| Ce | 6.54 | 3.17 | Mn | 6.54 | 2.64 | Ta | 40.76 | 2.82 |
| Cf | 6.54 | 2.95 | Mo | 28.18 | 2.72 | Tb | 3.52 | 3.07 |
| Cl | 142.57 | 3.52 | N | 38.95 | 3.26 | Tc | 24.16 | 2.67 |
| Cm | 6.54 | 2.96 | Na | 251.62 | 2.80 | Te | 286.85 | 3.77 |
| Co | 7.05 | 2.56 | Nb | 29.69 | 2.82 | Th | 13.08 | 3.03 |
| Cr | 7.55 | 2.85 | Nd | 5.03 | 3.19 | Ti | 8.56 | 2.83 |
| Cs | 22.65 | 4.02 | Ne | 21.14 | 2.89 | Tl | 342.21 | 3.87 |
| Cu | 2.52 | 3.11 | Ni | 7.55 | 2.53 | Tm | 3.02 | 3.01 |
| Dy | 3.52 | 3.05 | No | 5.54 | 2.89 | U | 11.07 | 3.03 |
| Er | 3.52 | 3.02 | Np | 9.56 | 3.05 | V | 8.05 | 2.80 |
| Es | 6.04 | 2.94 | O | 48.16 | 3.03 | VOL_He | 10.22 | 2.58 |
| Eu | 4.03 | 3.11 | Os | 18.62 | 2.78 | W | 33.72 | 2.73 |
| F | 36.49 | 3.09 | P | 161.04 | 3.70 | Xe | 167.08 | 3.92 |
| Fe | 27.68 | 4.05 | Pa | 11.07 | 3.05 | Y | 36.23 | 2.98 |
| Fm | 6.04 | 2.93 | Pb | 333.65 | 3.83 | Yb | 114.74 | 2.99 |
| Fr | 25.16 | 4.37 | Pd | 24.16 | 2.58 | Zn | 27.68 | 4.05 |
| Ga | 201.30 | 3.91 | Pm | 4.53 | 3.16 | Zr | 34.72 | 2.78 |
| Gd | 4.53 | 3.00 | Po | 163.56 | 4.20 |  |  |  |

**S4.2. Geometric optimization methods**

The geometry optimization of MOF structures in this study was performed at the DFT level using the CASTEP^[11]^ module in Materials Studio 2024. All calculations were carried out with ultra-fine accuracy, employing the Perdew-Burke-Ernzerhof (PBE) exchange-correlation functional within the generalized gradient approximation (GGA). To more accurately account for intermolecular interactions, dispersion corrections were applied using the DFT-D method proposed by Grimme. A Monkhorst–Pack scheme was used to generate the k-point grid, and ultrasoft pseudopotentials were generated on-the-fly (OTFG). The geometry optimization included both atomic positions and cell parameters to ensure structural stability at the lowest energy configuration.

**S4.3.** **Calculations of IAST selectivity**

The single-component adsorption isotherms of CH_4_ and N_2_ fitted using the Dual-site Langmuir Freundlich (DSLF) model, the formula of the DSLF model as follows:

$N=N_{A}^{\max}\frac{b_{A}p^{V_{A}}}{1+b_{A}p^{V_{A}}}+N_{B}^{\max}\frac{b_{B}p^{V_{B}}}{1+b_{B}p^{V_{B}}}$ (8)

In this equation, *N* is the adsorption capacity of the adsorbent (cm^3^ g^-1^); *p* is the adsorption pressure (bar); $\text{N}_{\text{A}}^{\text{max}}$ and $\text{N}_{\text{B}}^{\text{max}}$ are the saturation adsorption capacity at two different adsorption sites (cm^3^ g^-1^); *b_A_* and *b_B_* are the adsorption affinities at A and B site (bar^-1^), respectively; and *V_A_* and *V_B_* represent the deviations from an ideal homogeneous surface, respectively.

The adsorption selectivity base on ideal adsorbed solution theory (IAST) developed by Myers and Prausnitz^[12]^ for CH_4_/N_2_ was calculated using the expression:

(9)

**S4.4. Calculations of separation potential**

The separation potential (△*Q*, mol L^-1^) is a combined metric, which incorporates· both uptake and selectivity,.which was firstly introduced by Rajamani Krishna.^[13]^ It is defined to evaluate the·separation performances in fixed bed adsorbers·for gas mixture. For a·CH_4_/N_2_ mixture·with·mole fractions *y*_CH4_, and *y*_N2_=1- *y*_CH4_, the gravimetric separation potential △*Q*, is calculated from IAST base on the following·equation:

$\Delta Q=(q_{\text{CH}_{4}}\frac{y_{\text{N}_{2}}}{y_{\text{CH}_{4}}})-q_{\text{N}_{2}}$ (10)

Where *q*_CH4_, and *q*_N2_, are CH_4_ and N_2_ uptake in the mixture, respectively, which are calculated based on IAST theory.

**S4.5. Calculations of adsorption heat**

For the calculation of the isosteric heats of adsorption of pure CH_4_ and N_2_ at infinite dilution ($Q_{\text{st}}^{0}$), configurational-bias Monte Carlo simulations in the canonical (NVT) ensemble were further performed using the revised Widom’s test particle method.^[14]^

**S4.6. Calculations of pore volume**

The pore volume of MOFs in this study was calculated using a helium-based thermodynamic method. The approach proposed by Myer and Monson^[15]^ estimates the pore volume through a potential-energy-based configurational integral at room temperature, using a simple Monte Carlo sampling technique over the entire material structure. Therefore, this method was adopted in our work and has also been widely applied in other studies ^[16-18]^.

**Section S5. GCMC simulation results**


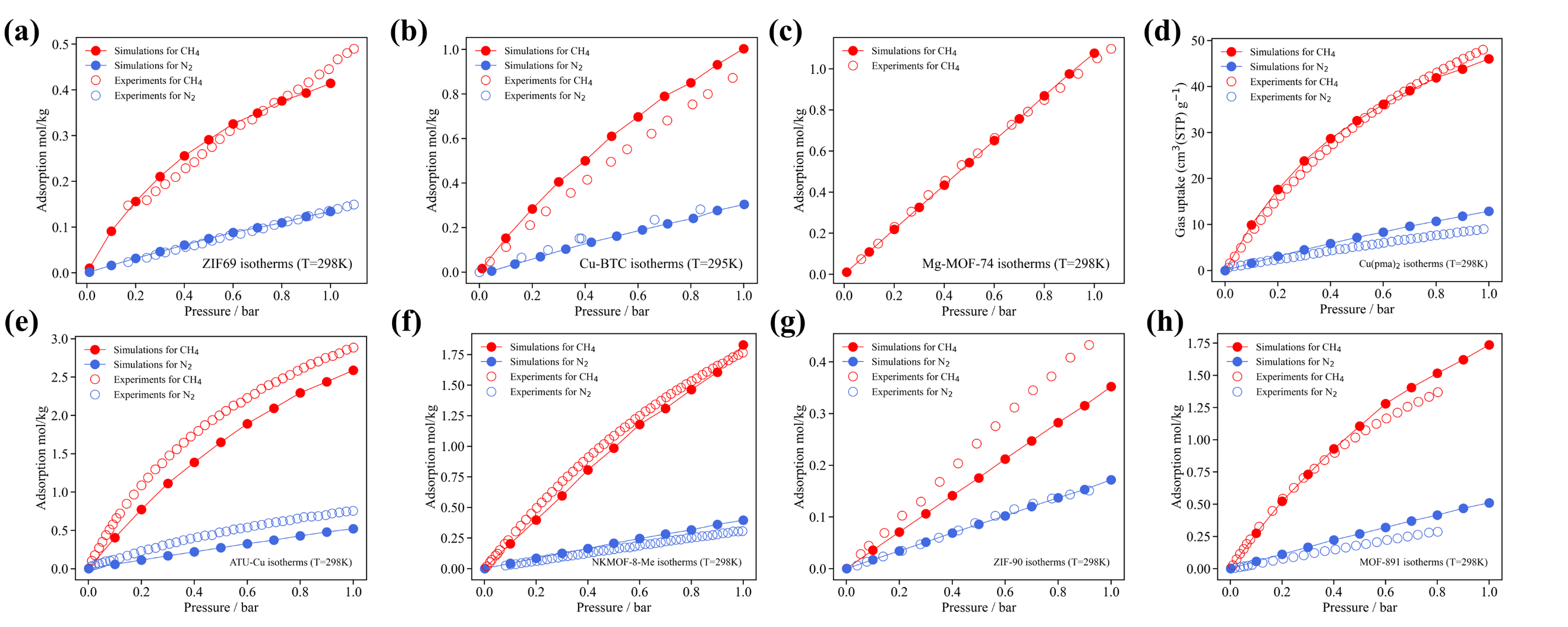


**Figure S6.** Comparison of the simulated adsorption isotherms of (a) ZIF69^[19]^ (b) Cu-BTC^[20, 21]^ (c) Mg-MOF-74^[22]^ (d) Cu(pma)_2_^[23]^ (e) ATU-Cu^[24]^ (f) NKMOF-8-Me^[25]^ (g) ZIF-90^[26]^ (h) MOF-891^[27]^ with experimental data.


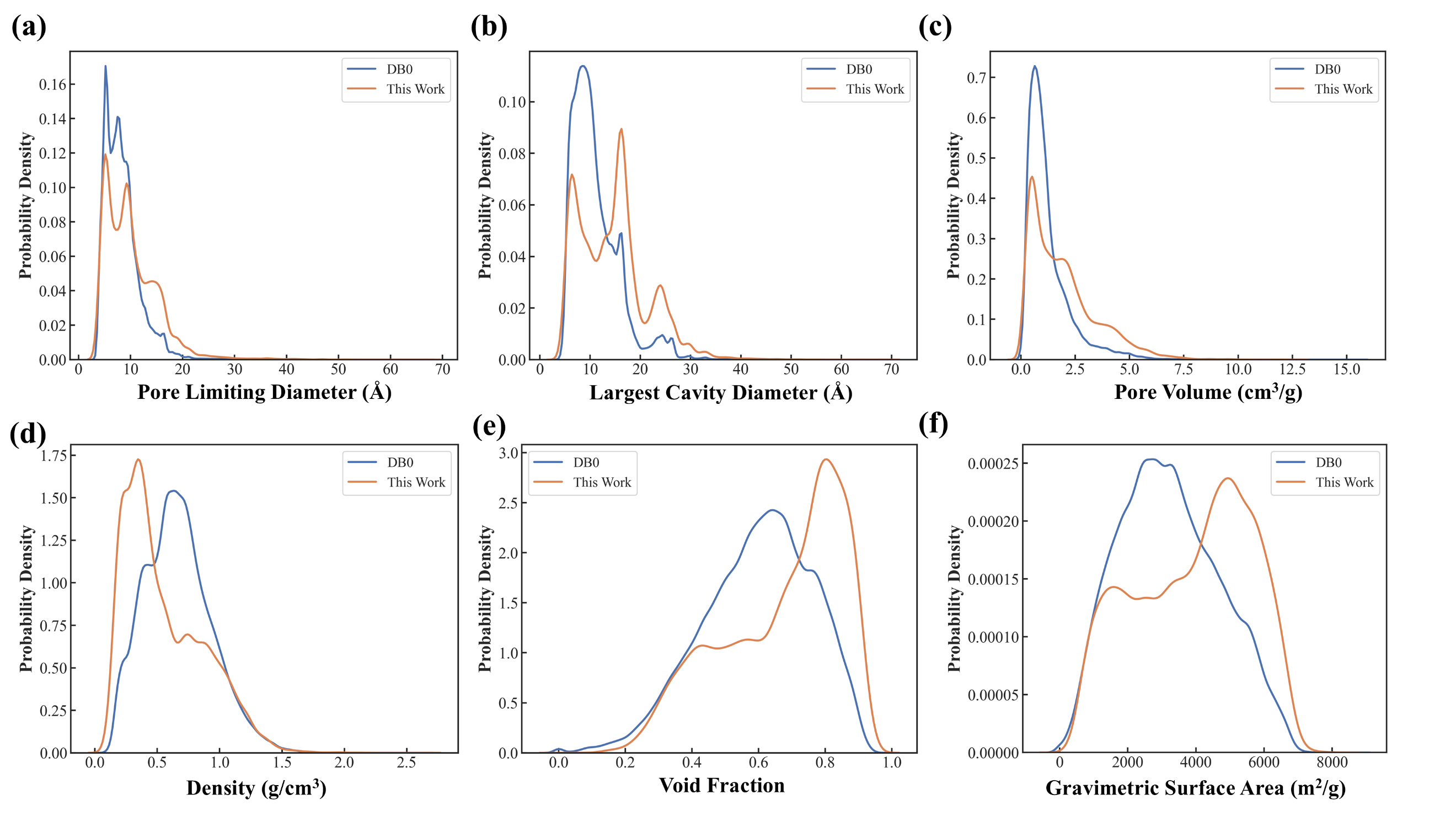


**Figure S7.** Common geometric parameters within the database in this work and DB0^[28]^: (a) pore limiting diameter; (b) largest cavity diameter; (c) density; (d) pore volume; (e) gravimetric surface area; and (f) porosity.


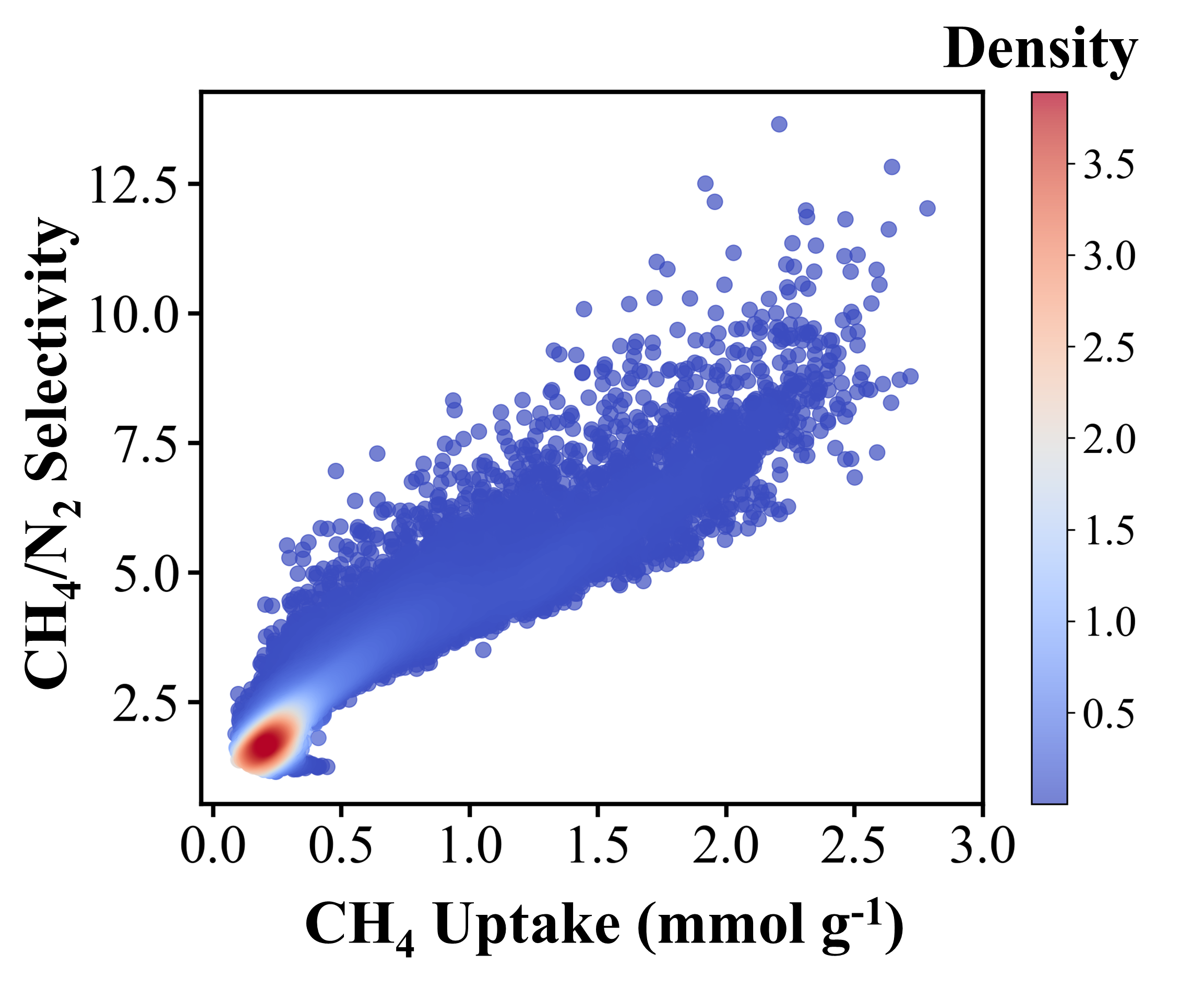


**Figure S8.** Scatter plot of CH_4_/N_2_ selectivity versus CH_4_ uptake for our database.

**Table S4.** Top 10 topologies in our database.

| Rank | Topology | Count | Percentage (%) |
| --- | --- | --- | --- |
| 1 | nbo | 12322 | 49.90 |
| 2 | fsc | 5784 | 23.42 |
| 3 | pcu | 3263 | 13.21 |
| 4 | fof | 568 | 2.30 |
| 5 | pts | 318 | 1.29 |
| 6 | bcu | 221 | 0.89 |
| 7 | tfz | 114 | 0.46 |
| 8 | jea | 87 | 0.35 |
| 9 | xbe | 67 | 0.27 |
| 10 | tfo | 55 | 0.22 |

**Table S5.** Top 10 MOFs for CH_4_/N_2_ separation in our database.

| Rank | Genotype of MOFs | S_XGBoost_ | S_GCMC_ |
| --- | --- | --- | --- |
| 1 | 1-34-0-18-17-2 | 13.03 | 13.65 |
| 2 | 1-34-0-18-11-2 | 12.49 | 12.83 |
| 3 | 1-32-10-12-10-2 | 11.73 | 12.51 |
| 4 | 1-32-10-12-10-2 | 11.73 | 12.15 |
| 5 | 1-30-0-18-18-2 | 10.93 | 12.03 |
| 6 | 1-34-0-18-2-2 | 11.00 | 11.99 |
| 7 | 1-34-0-18-2-2 | 11.00 | 11.86 |
| 8 | 1-34-0-18-3-2 | 11.26 | 11.82 |
| 9 | 1-34-0-18-3-2 | 11.26 | 11.62 |
| 10 | 1-32-5-12-5-2 | 11.15 | 11.35 |

**Section S6. Evaluation of machine learning and TAGA**

**Table S6.** Model evaluation metrics on the training set.

| ML Model | *R^2^* | MAE | SRCC |
| --- | --- | --- | --- |
| XGBoost | 0.98 | 0.14 | 0.98 |
| RF | 0.99 | 0.087 | 0.99 |
| SVM | 0.57 | 0.61 | 0.79 |
| LR | 0.29 | 0.92 | 0.59 |

**Table S7.** Model evaluation metrics on the test set.

| ML Model | *R^2^* | MAE | SRCC |
| --- | --- | --- | --- |
| XGBoost | 0.96 | 0.18 | 0.98 |
| RF | 0.95 | 0.18 | 0.98 |
| SVM | 0.57 | 0.61 | 0.79 |
| LR | 0.29 | 0.92 | 0.60 |

**Table S8.** XGBoost model 5-fold cross-validation evaluation metrics.

| Fold | Training Set  *R*^2^ MAE SRCC | | | Test Set  *R*^2^ MAE SRCC | | |
| --- | --- | --- | --- | --- | --- | --- |
| 1 | 0.98 | 0.14 | 0.98 | 0.96 | 0.18 | 0.98 |
| 2 | 0.98 | 0.14 | 0.98 | 0.97 | 0.17 | 0.98 |
| 3 | 0.98 | 0.14 | 0.98 | 0.96 | 0.18 | 0.98 |
| 4 | 0.98 | 0.14 | 0.98 | 0.96 | 0.17 | 0.98 |
| 5 | 0.98 | 0.14 | 0.98 | 0.96 | 0.17 | 0.98 |

**Table S9.** RF model 5-fold cross-validation evaluation metrics.

| Fold | Training Set  *R*^2^ MAE SRCC | | | Test Set  *R*^2^ MAE SRCC | | |
| --- | --- | --- | --- | --- | --- | --- |
| 1 | 0.99 | 0.09 | 0.99 | 0.95 | 0.18 | 0.98 |
| 2 | 0.99 | 0.09 | 0.99 | 0.96 | 0.18 | 0.98 |
| 3 | 0.99 | 0.09 | 0.99 | 0.95 | 0.19 | 0.97 |
| 4 | 0.99 | 0.09 | 0.99 | 0.96 | 0.18 | 0.98 |
| 5 | 0.99 | 0.09 | 0.99 | 0.95 | 0.18 | 0.98 |

**Table S10.** SVM model 5-fold cross-validation evaluation metrics.

| Fold | Training Set  *R*^2^ MAE SRCC | | | Test Set  *R*^2^ MAE SRCC | | |
| --- | --- | --- | --- | --- | --- | --- |
| 1 | 0.57 | 0.61 | 0.78 | 0.57 | 0.61 | 0.79 |
| 2 | 0.58 | 0.61 | 0.78 | 0.57 | 0.61 | 0.78 |
| 3 | 0.58 | 0.61 | 0.78 | 0.57 | 0.63 | 0.78 |
| 4 | 0.57 | 0.61 | 0.78 | 0.58 | 0.60 | 0.79 |
| 5 | 0.58 | 0.61 | 0.79 | 0.57 | 0.61 | 0.78 |

**Table S11.** LR model 5-fold cross-validation evaluation metrics.

| Fold | Training Set  *R*^2^ MAE SRCC | | | Test Set  *R*^2^ MAE SRCC | | |
| --- | --- | --- | --- | --- | --- | --- |
| 1 | 0.29 | 0.92 | 0.59 | 0.29 | 0.92 | 0.60 |
| 2 | 0.30 | 0.92 | 0.59 | 0.28 | 0.92 | 0.57 |
| 3 | 0.29 | 0.91 | 0.59 | 0.30 | 0.94 | 0.59 |
| 4 | 0.29 | 0.92 | 0.59 | 0.30 | 0.91 | 0.59 |
| 5 | 0.30 | 0.92 | 0.59 | 0.29 | 0.92 | 0.60 |

**Table S12.** Feature importance based on Mean(|SHAP|) from the XGBoost model.

| Feature Name | Mean(\|SHAP\|) |
| --- | --- |
| Topology | 0.91 |
| Linker 1 | 0.33 |
| Linker 2 | 0.27 |
| FG 1 | 0.16 |
| FG 2 | 0.14 |
| Node | 0.04 |


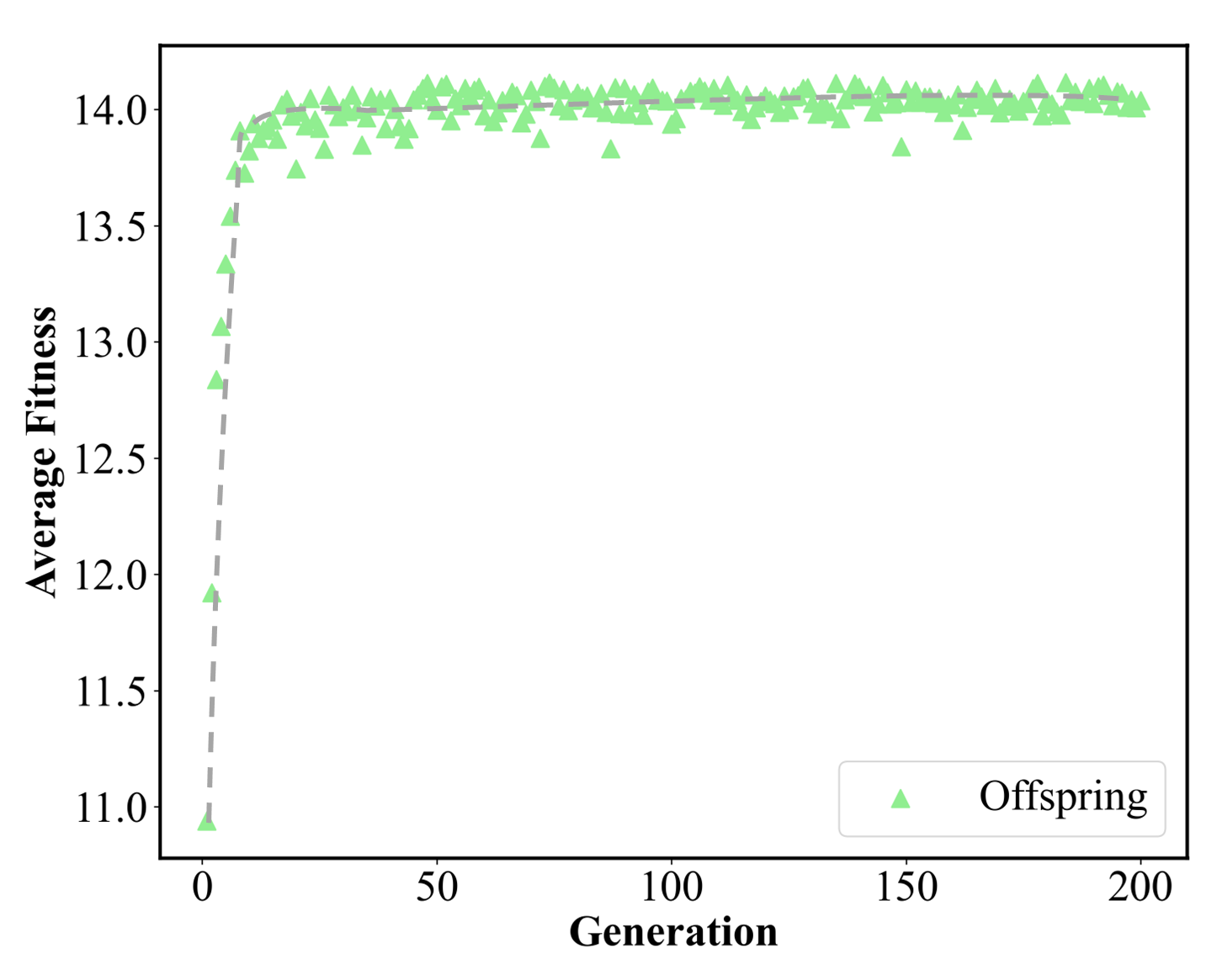


**Figure S9.** Scatter plot of average fitness versus generation number, with the gray dashed line indicating the overall trend.


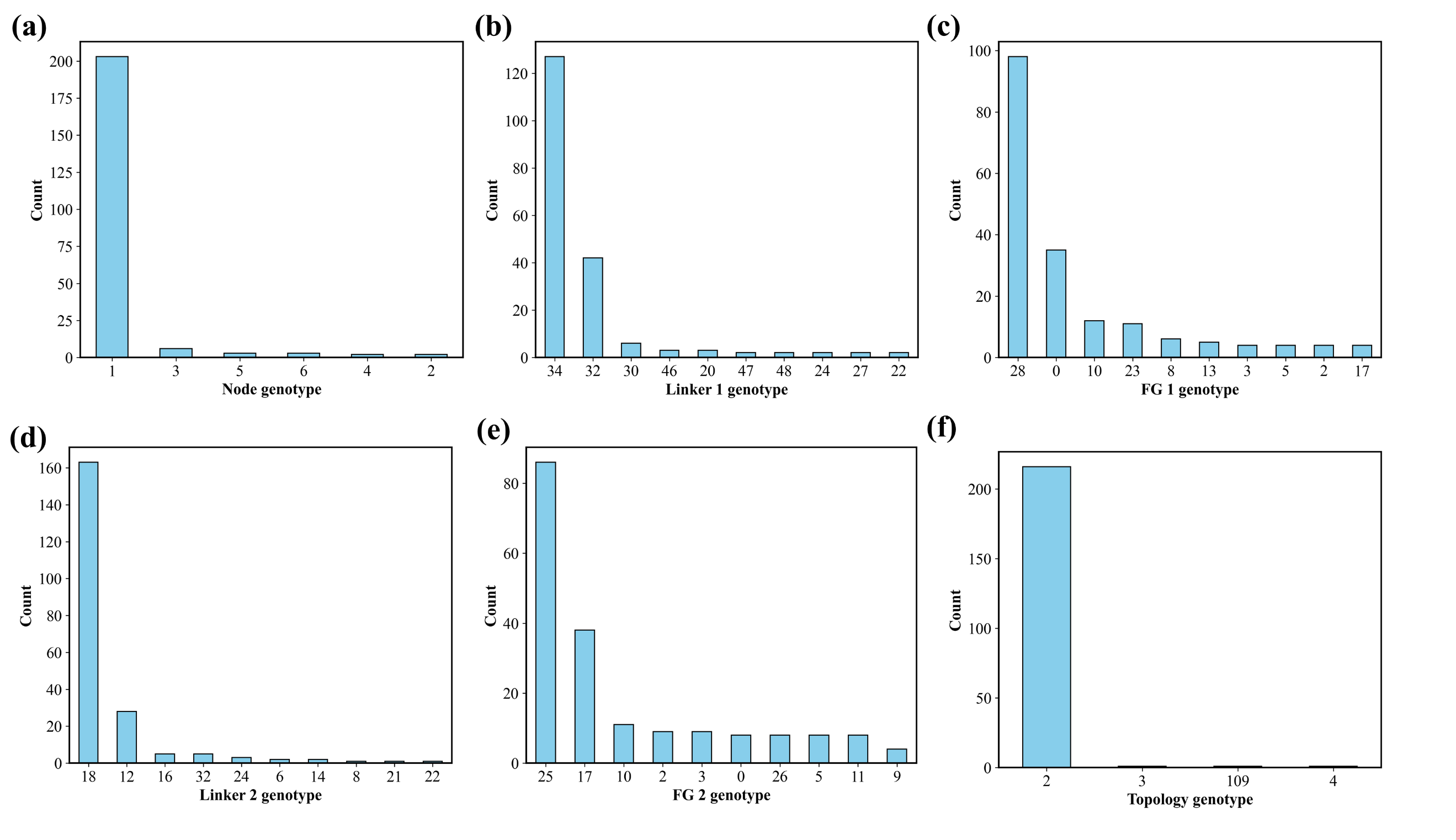


**Figure S10.** Distribution of Evolved genotypes after TAGA, (a) Node; (b) Linker 1; (c) FG 1; (d) Linker 2; (e) FG 2; (f) Topology.


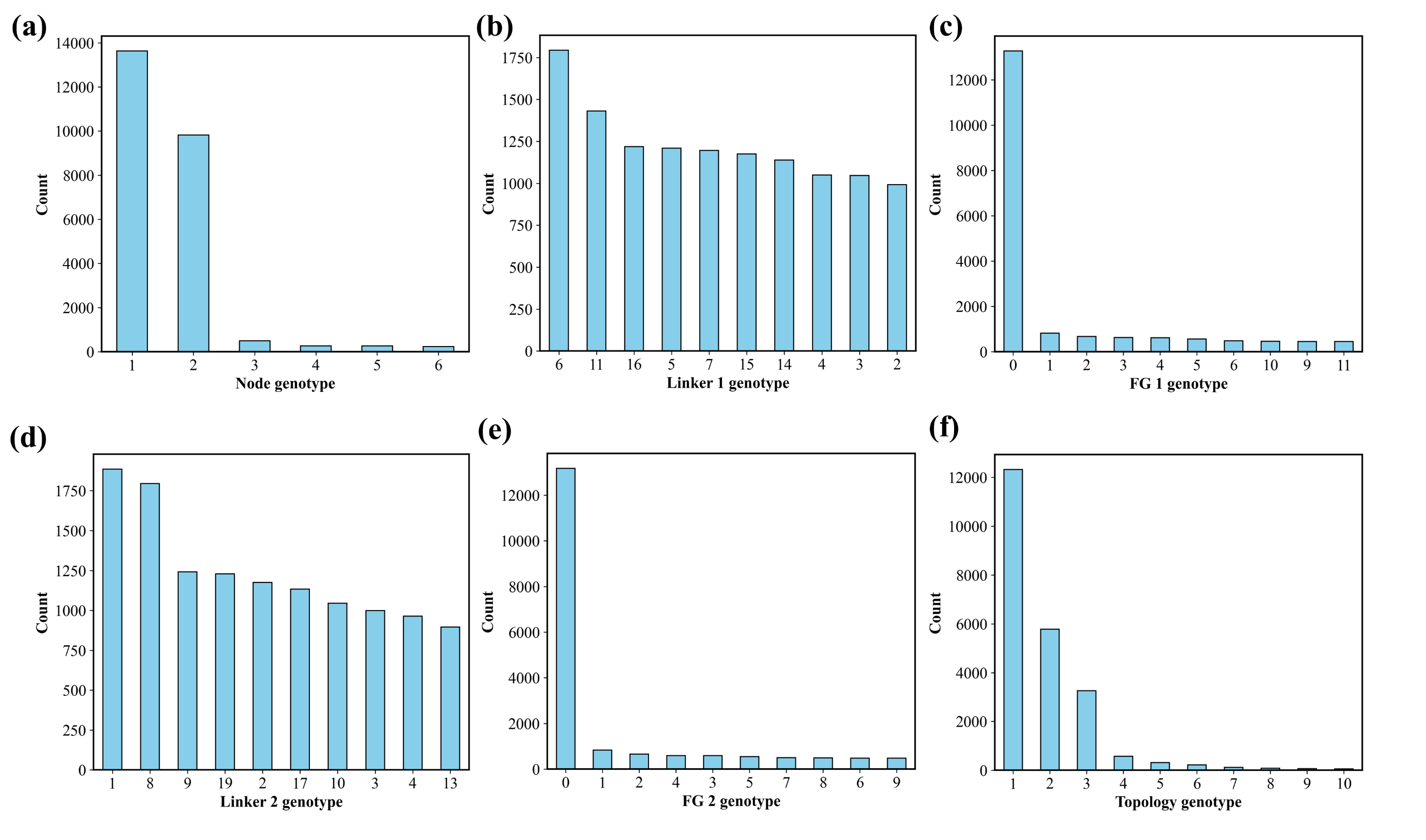


**Figure S11.** Distribution of Evolved genotypes before TAGA, (a) Node; (b) Linker 1; (c) FG 1; (d) Linker 2; (e) FG 2; (f) Topology.

**Section S7. Details of the Top-performance MOFs**


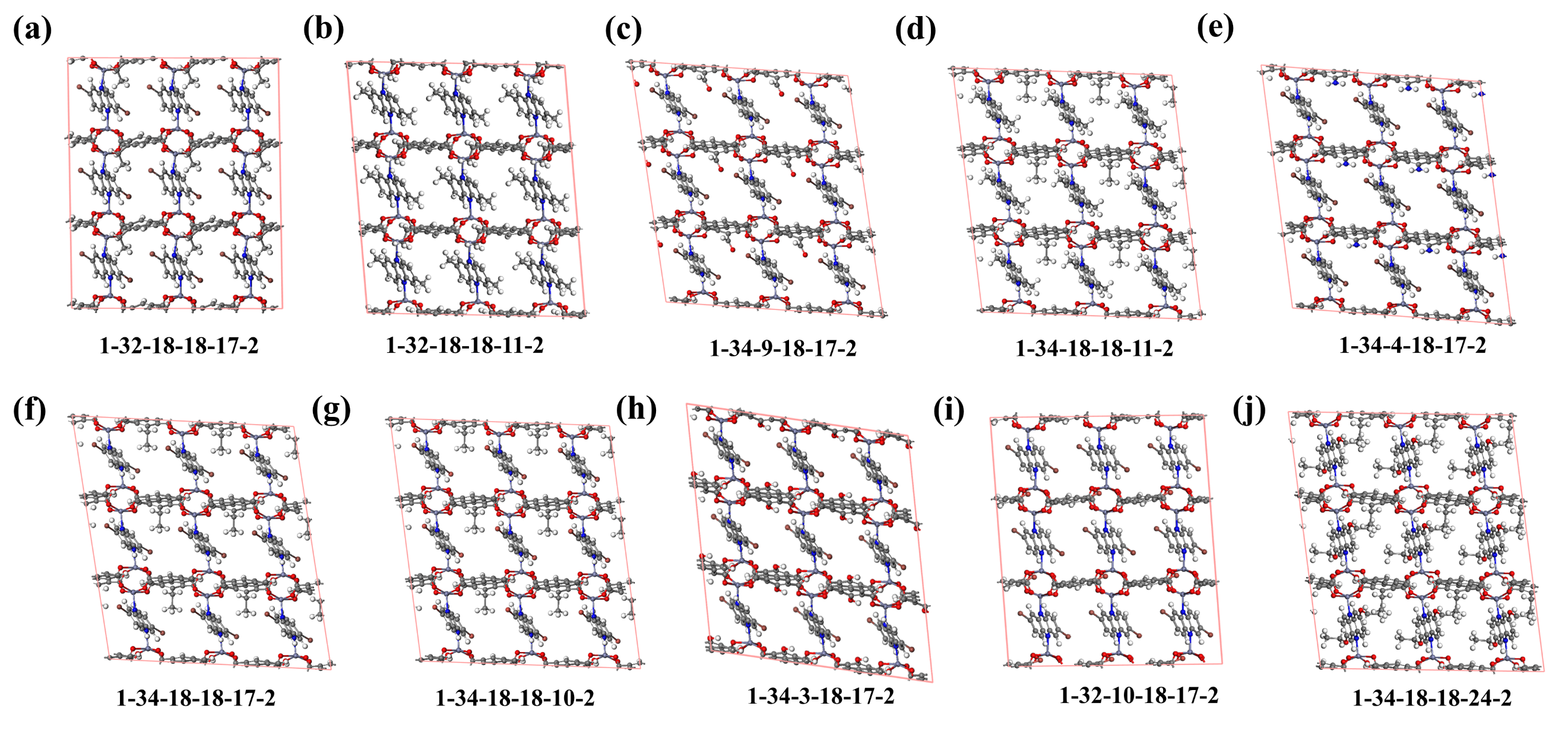


**Figure S12.** Crystal structures of the top 10 MOFs generated in this study with the highest CH_4_/N_2_ separation performance. The numerical sequence represents the genotype of the MOF.


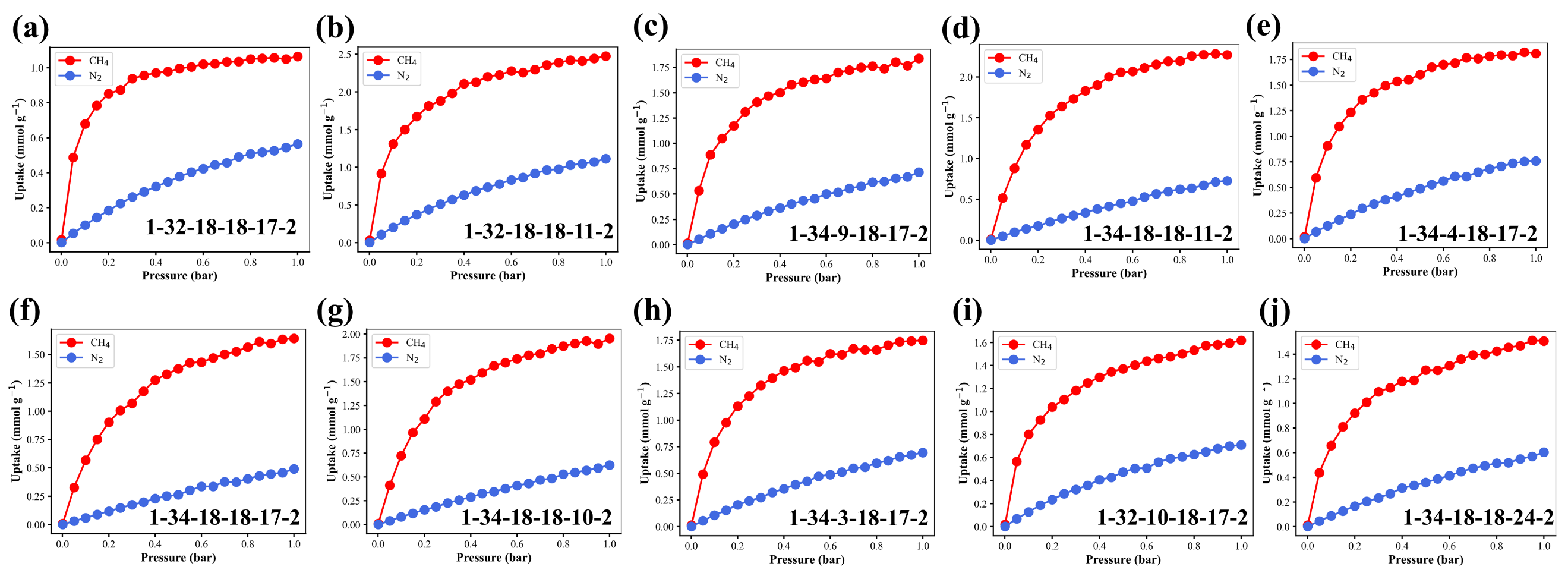


**Figure S13.** Simulated CH_4_ and N_2_ adsorption isotherms at 298 K for the top 10 MOFs, obtained from GCMC calculations. The numerical sequence represents the genotype of the MOF.

**Table S13.** Synthetic accessibility (SA) score^[29]^ and Synthetic complexity (SC) score^[30]^ of Top 10 generated MOFs.

| Chromosome of MOFs | Ligand | SMILES | SA score | SC score |
| --- | --- | --- | --- | --- |
| 1-32-18-18-17-2 | 1 | CCC1=C2C(=CC(=CC2=C(C=C1C(O)=O)C(O)=O)C(O)=O)C(O)=O | 2.44 | 3.42 |
|  | 2 | Ic1ccc2nc3cc(I)ccc3nc2c1 | 2.31 | 2.86 |
| 1-32-18-18-11-2 | 1 | CCC1=C2C(=CC(=CC2=C(C=C1C(O)=O)C(O)=O)C(O)=O)C(O)=O | 2.44 | 3.42 |
|  | 2 | Cc1ccc2nc3cc(C)ccc3nc2c1 | 1.81 | 2.86 |
| 1-34-9-18-17-2 | 1 | OC(=O)C1=CC(C(O)=O)=C2C=CC3=C(C(O)=O)C(C=O)=C(C(O)=O)C4=CC=C1C2=C34 | 2.75 | 3.58 |
|  | 2 | Ic1ccc2nc3cc(I)ccc3nc2c1 | 2.31 | 2.86 |
| 1-34-18-18-11-2 | 1 | CCC1=C(C(O)=O)C2=CC=C3C(=CC(C(O)=O)=C4C=CC(=C1C(O)=O)C2=C34)C(O)=O | 2.61 | 3.70 |
|  | 2 | Cc1ccc2nc3cc(C)ccc3nc2c1 | 1.81 | 2.86 |
| 1-34-4-18-17-2 | 1 | NC1=C(C(O)=O)C2=CC=C3C(=CC(C(O)=O)=C4C=CC(=C1C(O)=O)C2=C34)C(O)=O | 2.62 | 3.75 |
|  | 2 | Ic1ccc2nc3cc(I)ccc3nc2c1 | 2.31 | 2.86 |
| 1-34-18-18-17-2 | 1 | CCC1=C(C(O)=O)C2=CC=C3C(=CC(C(O)=O)=C4C=CC(=C1C(O)=O)C2=C34)C(O)=O | 2.61 | 3.70 |
|  | 2 | Ic1ccc2nc3cc(I)ccc3nc2c1 | 2.31 | 2.86 |
| 1-34-18-18-10-2 | 1 | CCC1=C(C(O)=O)C2=CC=C3C(=CC(C(O)=O)=C4C=CC(=C1C(O)=O)C2=C34)C(O)=O | 2.61 | 3.70 |
|  | 2 | Brc1ccc2nc3cc(Br)ccc3nc2c1 | 2.01 | 2.55 |
| 1-34-3-18-17-2 | 1 | OC(=O)C1=C(O)C(C(O)=O)=C2C=CC3=C(C(O)=O)C(O)=C(C(O)=O)C4=CC=C1C2=C34 | 2.46 | 3.65 |
|  | 2 | Ic1ccc2nc3cc(I)ccc3nc2c1 | 2.31 | 2.86 |
| 1-32-10-18-17-2 | 1 | O=C(O)c1cc(C(=O)O)c2c(Br)c(C(=O)O)cc(C(=O)O)c2c1 | 2.51 | 3.19 |
|  | 2 | Ic1ccc2nc3cc(I)ccc3nc2c1 | 2.31 | 2.86 |
| 1-34-18-18-24-2 | 1 | CCC1=C(C(O)=O)C2=CC=C3C(=CC(C(O)=O)=C4C=CC(=C1C(O)=O)C2=C34)C(O)=O | 2.61 | 3.70 |
|  | 2 | CCOc1ccc2nc3cc(OCC)ccc3nc2c1 | 1.91 | 2.86 |

**Table S14.** Machine Learning-Predicted Stability Metrics of the Top 10 generated MOFs.^[31,32]^

| Rank | Chromosome of MOFs | Thermal stability (℃) | Water stability probability |
| --- | --- | --- | --- |
| 1 | 1-32-18-18-17-2 | 406.80 | 0.55 |
| 2 | 1-32-18-18-11-2 | 436.80 | 0.48 |
| 3 | 1-34-9-18-17-2 | 413.30 | 0.56 |
| 4 | 1-34-18-18-11-2 | 455.00 | 0.46 |
| 5 | 1-34-4-18-17-2 | 399.40 | 0.52 |
| 6 | 1-34-18-18-17-2 | 401.40 | 0.50 |
| 7 | 1-34-18-18-10-2 | 388.70 | 0.53 |
| 8 | 1-34-3-18-17-2 | 412.10 | 0.58 |
| 9 | 1-32-10-18-17-2 | 448.20 | 0.60 |
| 10 | 1-34-18-18-24-2 | 411.80 | 0.33 |

**Table S15.** Performance and structure parameters of top 10 generated MOFs.

| Rank | Chromosome of MOFs | Uptake [mmol g^-1^]  CH_4_ N_2_ | | $Q_{\text{st}}^{0}$ [kJ mol^-1^]  CH_4_ N_2_ | | $\Delta Q_{\text{st}}^{0}$  [kJ mol^-1^] | Pore Volume  [cm^3^ g^-1^] | Porosity |
| --- | --- | --- | --- | --- | --- | --- | --- | --- |
| 1 | 1-32-18-18-17-2 | 0.97 | 0.06 | -28.66 | -21.52 | 7.14 | 0.12 | 0.23 |
| 2 | 1-32-18-18-11-2 | 2.14 | 0.14 | -28.13 | -21.36 | 6.77 | 0.21 | 0.31 |
| 3 | 1-34-9-18-17-2 | 1.54 | 0.10 | -28.44 | -21.81 | 6.62 | 0.16 | 0.29 |
| 4 | 1-34-18-18-11-2 | 1.91 | 0.13 | -27.15 | -20.82 | 6.33 | 0.21 | 0.29 |
| 5 | 1-34-4-18-17-2 | 1.58 | 0.11 | -27.52 | -20.95 | 6.57 | 0.19 | 0.34 |
| 6 | 1-34-18-18-17-2 | 1.36 | 0.10 | -28.27 | -21.87 | 6.41 | 0.14 | 0.25 |
| 7 | 1-34-18-18-10-2 | 1.61 | 0.12 | -27.14 | -20.80 | 6.34 | 0.19 | 0.30 |
| 8 | 1-34-3-18-17-2 | 1.47 | 0.11 | -27.89 | -21.56 | 6.33 | 0.16 | 0.30 |
| 9 | 1-32-10-18-17-2 | 1.32 | 0.10 | -28.33 | -21.50 | 6.83 | 0.14 | 0.29 |
| 10 | 1-34-18-18-24-2 | 1.21 | 0.10 | -28.53 | -21.94 | 6.60 | 0.16 | 0.23 |

**Table S16.** Fitting parameters of the DSLF model for the single-component adsorption isotherms of CH_4_ and N_2_ in the top 10 MOFs at 298 K.

| Chromosome of MOFs | Gas | $\text{N}_{\text{A}}^{\text{max}} [$mol L^-1^] | $\text{b}_{\text{A}} [$bar^-1^] | *ν*_A_ | $\text{N}_{\text{B}}^{\text{max}} [$mol L^-1^] | $\text{b}_{\text{B}} [$bar^-1^] | *ν*_B_ |
| --- | --- | --- | --- | --- | --- | --- | --- |
| 1-32-18-18-17-2 | CH_4_ | 2.18511 | 15.14550 | 0.99310 | 0.01687 | 1105616.60400 | 10.96268 |
|  | N_2_ | 0.89670 | 1.33524 | 1.37206 | 0.94699 | 1.58267 | 0.93123 |
| 1-32-18-18-11-2 | CH_4_ | 2.28475 | 28.95574 | 1.06397 | 1.77395 | 3.97206 | 1.34495 |
|  | N_2_ | 3.10484 | 1.07513 | 1.00329 | 0.02306 | 621471.37590 | 33.59397 |
| 1-34-9-18-17-2 | CH_4_ | 3.57323 | 4.89765 | 0.97933 | 0.41374 | 2884920.20400 | 4.82579 |
|  | N_2_ | 1.31048 | 0.02761 | 495.00912 | 3.22696 | 0.66239 | 1.00610 |
| 1-34-18-18-11-2 | CH_4_ | 0.07161 | 1545836.15500 | 18.13068 | 3.58689 | 6.34532 | 1.08422 |
|  | N_2_ | 0.03381 | 848921.70680 | 30.31044 | 3.06776 | 0.46465 | 1.02053 |
| 1-34-4-18-17-2 | CH_4_ | 3.55984 | 8.87624 | 1.01340 | 0.10055 | 2592948.04600 | 23.41345 |
|  | N_2_ | 0.20850 | 4.89478 | 4.84821 | 1.89404 | 1.79501 | 1.10934 |
| 1-34-18-18-17-2 | CH_4_ | 3.67747 | 3.89592 | 0.99310 | 0.07389 | 2882741.12500 | 14.73614 |
|  | N_2_ | 0.02147 | 1120977.78000 | 22.99938 | 3.13384 | 0.37812 | 1.01075 |
| 1-34-18-18-10-2 | CH_4_ | 0.03661 | 963374.95700 | 45.45213 | 3.63116 | 6.18368 | 1.10992 |
|  | N_2_ | 2.75943 | 0.52474 | 1.02682 | 0.09984 | 1.63681 | 5.62400 |
| 1-34-3-18-17-2 | CH_4_ | 0.02631 | 734215.30800 | 89.21465 | 3.77330 | 6.54607 | 1.00703 |
|  | N_2_ | 0.02661 | 356373.81410 | 86.14532 | 3.07215 | 0.71449 | 1.01839 |
| 1-32-10-18-17-2 | CH_4_ | 0.55134 | 1371042.51000 | 4.27040 | 3.97702 | 2.30240 | 0.77627 |
|  | N_2_ | 0.02221 | 1431018.29900 | 32.77123 | 2.99451 | 0.93359 | 0.97861 |
| 1-34-18-18-24-2 | CH_4_ | 0.12302 | 5.53321 | 6.74142 | 2.44625 | 5.64374 | 0.94827 |
|  | N_2_ | 2.15457 | 0.66199 | 1.03063 | 0.03300 | 1.07507 | 302.56781 |

**Table S17.** Experimentally reported MOFs for CH_4_/N_2_ separation.

| The names of MOFs | CH_4_ uptake [mmol g^-1^] | IAST selectivity | Ref. |
| --- | --- | --- | --- |
| CoNi-DAB | 1.08 | 19.1 | ^[33]^ |
| Ni(ina)_2_ | 1.82 | 15.8 | ^[34]^ |
| Al-CDC | 1.43 | 13.1 | ^[35]^ |
| Co_3_(C4O4)_2_(OH)_2_ | 0.40 | 12.5 | ^[36]^ |
| CAU-21-BPDC | 0.99 | 11.9 | ^[37]^ |
| CoNi(pyz-NH_2_) | 0.82 | 11.9 | ^[38]^ |
| Cu(pma)_2_ | 1.96 | 11.5 | ^[23]^ |
| SBMOF-1 | 0.92 | 11.5 | ^[39]^ |
| STAM-1 | 0.63 | 11.1 | ^[40]^ |
| ATU-Cu | 2.90 | 9.7 | ^[41]^ |
| ROD-9 | 0.77 | 9.1 | ^[42]^ |
| NKMOF-8-Me | 1.76 | 9.0 | ^[25]^ |
| MOF-891 | 1.34 | 7.8 | ^[27]^ |
| Ni-MA-BPY | 1 | 7.4 | ^[43]^ |
| ZIF-94 | 1.5 | 7.0 | ^[44]^ |
| Ni(Oac)_2_L | 1.15 | 7.0 | ^[45]^ |
| Cu(hfipbb)(H2hfipbb)_0.5_ | 0.47 | 6.9 | ^[46]^ |
| Ni-BTC | 1.25 | 6.3 | ^[47]^ |
| Cu(OTf)_2_ | 0.25 | 4.8 | ^[48]^ |
| [Cu(Me-4py-trz-ia)] | 1.12 | 4.2 | ^[49]^ |
| ZIF-69 | 0.5 | 3.0 | ^[50]^ |
| Mg-MOF-74 | 1.66 | 1.5 | ^[51]^ |
| Co-MOF-74 | 1.91 | 3.2 | ^[51]^ |

**References**

[1] T. Yan, Z. Bi, D. Liu, X. Zhang, G. Lu, Q. Yang, A Self-Evolutionary Methodology for Reverse Design of Novel MOFs. *J. Phys. Chem. A* **2022**, *126*, 8476-8486.

[2] X. Su, X. Yan, C. L. Tsai, Linear regression. *Wiley Interdiscip. Rev.: Comput. Stat.* **2012**, *4*, 275-294.

[3] A. Mammone, M. Turchi, N. Cristianini, Support vector machines. *Wiley Interdiscip. Rev.: Comput. Stat.* **2009**, *1*, 283-289.

[4] H. Liang, K. Jiang, T. A. Yan, G. H. Chen, XGBoost: An Optimal Machine Learning Model with Just Structural Features to Discover MOF Adsorbents of Xe/Kr. *ACS Omega* **2021**, *6*, 9066-9076.

[5] M. Belgiu, L. Drăguţ, Random forest in remote sensing: A review of applications and future directions. *ISPRS J. Photogrammetry Remote Sens.* **2016**, *114*, 24-31.

[6] W. Li, Y. Situ, L. Ding, Y. Chen, Q. Yang, MOF-GRU: A MOFid-Aided Deep Learning Model for Predicting the Gas Separation Performance of Metal-Organic Frameworks. *ACS Appl. Mater. Interfaces* **2023**, *15*, 59887-59894.

[7] H. Tang, Q. Xu, M. Wang, J. Jiang, Rapid Screening of Metal-Organic Frameworks for Propane/Propylene Separation by Synergizing Molecular Simulation and Machine Learning. *ACS Appl. Mater. Interfaces* **2021**, *13*, 53454-53467.

[8] J. D. Rodriguez, A. Perez, J. A. Lozano, Sensitivity Analysis of k-Fold Cross Validation in Prediction Error Estimation. *IEEE Trans. Pattern Anal. Mach. Intell.* **2010**, *32*, 569-575.

[9] Lundberg, S. M., S.-I. Lee, A unified approach to interpreting model predictions. Advances in neural information processing systems. **2017**, 30.

[10] S. M. Lundberg, G. Erion, H. Chen, A. DeGrave, J. M. Prutkin, B. Nair, R. Katz, J. Himmelfarb, N. Bansal, S. I. Lee, From Local Explanations to Global Understanding with Explainable AI for Trees. *Nat Mach Intell* **2020**, *2*, 56-67.

[11] M. D. Segall, P. J. D. Lindan, M. J. Probert, C. J. Pickard, P. J. Hasnip, S. J. Clark, M. C. Payne, First-principles simulation: ideas, illustrations and the CASTEP code. *J. Phys.: Condens. Matter* **2002**, *14*, 2717-2744.

[12] A. L. Myers, J. M. Prausnitz, Thermodynamics of mixed-gas adsorption. *AIChE J.* **1965**, *11*, 121-127.

[13] R. Krishna, Screening metal–organic frameworks for mixture separations in fixed-bed adsorbers using a combined selectivity/capacity metric. *RSC Advances* **2017**, *7*, 35724-35737.

[14] T. Vlugt, E. García-Pérez, D. Dubbeldam, S. Ban, S. Calero, Computing the heat of adsorption using molecular simulations: the effect of strong Coulombic interactions. *J. Chem. Theory Comput.* **2008**, *4*, 1107-1118.

[15] A. L. Myers, P. A. Monson, Adsorption in porous materials at high pressure: theory and experiment. *Langmuir* **2002**, *18*, 10261-10273.

[16] Z. Li, Y. Zhang, B. Liu, G. Chen, B. Smit, Multilevel screening of computation‐ready, experimental metal‐organic frameworks for natural gas purification. *AIChE J.* **2021**, *67*, e17279.

[17] J. Camp, V. Stavila, M. D. Allendorf, D. Prendergast, M. Haranczyk, Critical factors in computational characterization of hydrogen storage in metal–organic frameworks. *J. Phys. Chem. C* **2018**, *122*, 18957-18967.

[18] S. Demir, N. Bilgin, H. M. Cepni, H. Furukawa, F. Yilmaz, C. Altintas, S. Keskin, Enhanced water stability and high CO_2_ storage capacity of a Lewis basic sites-containing zirconium metal–organic framework. *Dalton Trans.* **2021**, *50*, 16587-16592.

[19] R. Banerjee, H. Furukawa, D. Britt, C. Knobler, M. O'Keeffe, O. M. Yaghi, Control of Pore Size and Functionality in Isoreticular Zeolitic Imidazolate Frameworks and their Carbon Dioxide Selective Capture Properties. *J. Am. Chem. Soc.* **2009**, *131*, 3875-7.

[20] H. S. Koh, M. K. Rana, A. G. Wong-Foy, D. J. Siegel, Predicting Methane Storage in Open-Metal-Site Metal–Organic Frameworks. *J. Phys. Chem. C* **2015**, *119*, 13451-13458.

[21] G. Lei, G. Xi, Z. Liu, Q. Li, H. Cheng, H. Liu, Enhancing selective adsorption of CO_2_ through encapsulating FeTPPs into Cu-BTC. *Chem. Eng. J.* **2023**, *461*, 141977.

[22] Z. Bao, L. Yu, Q. Ren, X. Lu, S. Deng, Adsorption of CO_2_ and CH_4_ on a magnesium-based metal organic framework. *J. Colloid Interface Sci.* **2011**, *353*, 549-56.

[23] M. Chang, Y. Li, M. Tong, J. Yang, T. Ma, Y. Wang, J. Zheng, Synergistic Binding Sites in a Robust and Scalable Metal–Organic Framework for Record CH_4_ Capture. *Small* **2025**, *21*, 2412121.

[24] Z. Niu, X. Cui, T. Pham, P. C. Lan, H. Xing, K. A. Forrest, L. Wojtas, B. Space, S. Ma, A Metal–Organic Framework Based Methane Nano‐trap for the Capture of Coal‐Mine Methane. *Angew. Chem., Int. Ed.* **2019**, *58*, 10138-10141.

[25] M. Chang, F. Wang, Y. Wei, Q. Yang, J. X. Wang, D. Liu, J. F. Chen, Separation of CH_4_/N_2_ by an ultra‐stable metal–organic framework with the highest breakthrough selectivity. *AIChE J.* **2022**, *68*, e17794.

[26] Q. Shi, J. Wang, H. Shang, H. Bai, Y. Zhao, J. Yang, J. Dong, J. Li, Effective CH_4_ enrichment from N_2_ by SIM-1 via a strong adsorption potential SOD cage. *Sep. Purif. Technol.* **2020**, *230*, 115850.

[27] P. T. K. Nguyen, H. T. D. Nguyen, H. Q. Pham, J. Kim, K. E. Cordova, H. Furukawa, Synthesis and Selective CO_2_ Capture Properties of a Series of Hexatopic Linker-Based Metal–Organic Frameworks. *Inorg. Chem.* **2015**, *54*, 10065-10072.

[28] J. Burner, J. Luo, A. White, A. Mirmiran, O. Kwon, P. G. Boyd, S. Maley, M. Gibaldi, S. Simrod, V. Ogden, T. K. Woo, ARC–MOF: A Diverse Database of Metal-Organic Frameworks with DFT-Derived Partial Atomic Charges and Descriptors for Machine Learning. *Chem. Mater.* **2023**, *35*, 900-916.

[29] S. Chen, Y. Jung, Estimating the synthetic accessibility of molecules with building block and reaction-aware SAScore. *J. Cheminf.* **2024**, *16*, 83.

[30] C. W. Coley, L. Rogers, W. H. Green, K. F. Jensen, modeling, SCScore: synthetic complexity learned from a reaction corpus. *J. Chem. Inf. Model.* **2018**, *58*, 252-261.

[31] A. Nandy, C. Duan, H. J. Kulik, Using machine learning and data mining to leverage community knowledge for the engineering of stable metal–organic frameworks. *J. Am. Chem. Soc.* **2021**, *143*, 17535-17547.

[32] G. G. Terrones, S.-P. Huang, M. P. Rivera, S. Yue, A. Hernandez, H. J. Kulik, Metal–organic framework stability in water and harsh environments from data-driven models trained on the diverse WS24 data set. *J. Am. Chem. Soc.* **2024**, *146*, 20333-20348.

[33] Y.-L. Zhao, X. Bai, X. Zhang, Z.-Y. Han, J.-R. Li, Record high CH_4_/N_2_ adsorption separation selectivity in a scalable metal-organic framework. *Science bulletin* **2025**, *70*, 1215-1218.

[34] S. M. Wang, M. Shivanna, Q. Y. Yang, Nickel‐Based Metal–Organic Frameworks for Coal‐Bed Methane Purification with Record CH_4_/N_2_ Selectivity. *Angew. Chem., Int. Ed.* **2022**, *61*, e202201017.

[35] M. Chang, Y. Zhao, D. Liu, J. Yang, J. Li, C. Zhong, Methane-trapping metal–organic frameworks with an aliphatic ligand for efficient CH_4_/N_2_ separation. *Sustainable Energy & Fuels* **2020**, *4*, 138-142.

[36] L. Li, L. Yang, J. Wang, Z. Zhang, Q. Yang, Y. Yang, Q. Ren, Z. Bao, Highly efficient separation of methane from nitrogen on a squarate‐based metal‐organic framework. *AIChE J.* **2018**, *64*, 3681-3689.

[37] D. Lv, Y. Wu, J. Chen, Y. Tu, Y. Yuan, H. Wu, Y. Chen, B. Liu, H. Xi, Z. Li, Improving CH_4_/N_2_ selectivity within isomeric Al‐based MOFs for the highly selective capture of coal‐mine methane. *AIChE J.* **2020**, *66*, e16287.

[38] P. Guo, Y. Ying, D. Liu, One Scalable and Stable Metal–Organic Framework for Efficient Separation of CH_4_/N_2_ Mixture. *ACS Appl. Mater. Interfaces* **2024**, *16*, 7338-7344.

[39] M. Chang, J. Ren, Q. Yang, D. Liu, A robust calcium-based microporous metal-organic framework for efficient CH_4_/N_2_ separation. *Chem. Eng. J.* **2021**, *408*, 127294.

[40] M. Chang, Y. Zhao, Q. Yang, D. J. A. O. Liu, Microporous metal–organic frameworks with hydrophilic and hydrophobic pores for efficient separation of CH_4_/N_2_ mixture. *Acs Omega* **2019**, *4*, 14511-14516.

[41] Z. Niu, X. Cui, T. Pham, P. C. Lan, H. Xing, K. A. Forrest, L. Wojtas, B. Space, S. J. A. C. I. E. Ma, A metal–organic framework based methane nano‐trap for the capture of coal‐mine methane. *Angew. Chem., Int. Ed.* **2019**, *58*, 10138-10141.

[42] R.-J. Li, M. Li, X.-P. Zhou, S. W. Ng, M. O'Keeffe, D. Li, ROD-8, a rod MOF with a pyrene-cored tetracarboxylate linker: framework disorder, derived nets and selective gas adsorption. *CrystEngComm* **2014**, *16*, 6291-6295.

[43] X.-W. Liu, Y.-M. Gu, T.-J. Sun, Y. Guo, X.-L. Wei, S.-S. Zhao, S.-D. Wang, Water Resistant and Flexible MOF Materials for Highly Efficient Separation of Methane from Nitrogen. *Ind. Eng. Chem. Res.* **2019**, *58*, 20392-20400.

[44] Z. Huang, P. Hu, J. Liu, F. Shen, Y. Zhang, K. Chai, Y. Ying, C. Kang, Z. Zhang, H. Ji, Enhancing CH_4_/N_2_ separation performance within aluminum-based Metal-Organic Frameworks: Influence of the pore structure and linker polarity. *Sep. Purif. Technol.* **2022**, *286*, 120446.

[45] C. E. Kivi, B. S. Gelfand, H. Dureckova, H. T. K. Ho, C. Ma, G. K. H. Shimizu, T. K. Woo, D. Song, 3D porous metal–organic framework for selective adsorption of methane over dinitrogen under ambient pressure. *Chem. Commun.* **2018**, *54*, 14104-14107.

[46] X. Wu, B. Yuan, Z. Bao, S. Deng, Adsorption of carbon dioxide, methane and nitrogen on an ultramicroporous copper metal–organic framework. *J. Colloid Interface Sci.* **2014**, *430*, 78-84.

[47] X. Jia, N. Yuan, L. Wang, J. Yang, J. Li, (CH_3_) 2NH‐assisted synthesis of high‐purity Ni‐HKUST‐1 for the adsorption of CO_2_, CH_4_, and N_2_. *Eur. J. Inorg. Chem.* **2018**, *2018*, 1047-1052.

[48] X. Wang, L. Li, J. Yang, J. Li, CO_2_/CH_4_ and CH_4_/N_2_ separation on isomeric metal organic frameworks. *Chin. J. Chem. Eng.* **2016**, *24*, 1687-1694.

[49] J. Möllmer, M. Lange, A. Möller, C. Patzschke, K. Stein, D. Lässig, J. Lincke, R. Gläser, H. Krautscheid, R. Staudt, Pure and mixed gas adsorption of CH_4_ and N_2_ on the metal–organic framework Basolite® A100 and a novel copper-based 1,2,4-triazolyl isophthalate MOF. *J. Mater. Chem.* **2012**, *22*, 10274.

[50] B. Liu, B. Smit, Molecular simulation studies of separation of CO_2_/N_2_, CO_2_/CH_4_, and CH_4_/N_2_ by ZIFs. *J. Phys. Chem. C* **2010**, *114*, 8515-8522.

[51] L. Li, J. Yang, J. Li, Y. Chen, J. Li, Separation of CO_2_/CH_4_ and CH_4_/N_2_ mixtures by M/DOBDC: A detailed dynamic comparison with MIL-100(Cr) and activated carbon. *Microporous Mesoporous Mater.* **2014**, *198*, 236-246.
